# A System for the Detection of Persons in Intelligent Buildings Using Camera Systems—A Comparative Study

**DOI:** 10.3390/s20123558

**Published:** 2020-06-23

**Authors:** Miroslav Schneider, Zdenek Machacek, Radek Martinek, Jiri Koziorek, Rene Jaros

**Affiliations:** Department of Cybernetics and Biomedical Engineering, Faculty of Electrical Engineering and Computer Science, VSB–Technical University of Ostrava, 17. listopadu 15, 708 33 Ostrava, Czech Republic; miroslav.schneider@vsb.cz (M.S.); zdenek.machacek@vsb.cz (Z.M.); radek.martinek@vsb.cz (R.M.); jiri.koziorek@vsb.cz (J.K.)

**Keywords:** object detection, person detection, motion detection, background, static background, dynamic background

## Abstract

This article deals with the design and implementation of a prototype of an efficient Low-Cost, Low-Power, Low Complexity–hereinafter (L-CPC) an image recognition system for person detection. The developed and presented methods for processing, analyzing and recognition are designed exactly for inbuilt devices (e.g., motion sensor, identification of property and other specific applications), which will comply with the requirements of intelligent building technologies. The paper describes detection methods using a static background, where, during the search for people, the background image field being compared does not change, and a dynamic background, where the background image field is continually adjusted or complemented by objects merging into the background. The results are compared with the output of the Horn-Schunck algorithm applied using the principle of optical flow. The possible objects detected are subsequently stored and evaluated in the actual algorithm described. The detection results, using the change detection methods, are then evaluated using the Saaty method in order to determine the most successful configuration of the entire detection system. Each of the configurations used was also tested on a video sequence divided into a total of 12 story sections, in which the normal activities of people inside the intelligent building were simulated.

## 1. Introduction

Camera systems are increasingly found in the interior of not only intelligent buildings. These systems are most often used for obtaining a video signal with the aim of recording or online streaming to the control room of the security service, that is, in order to protect people or property, which is the subject of References [1,2]. However, such a video signal can be used for detection or identification of persons present in the scene being captured after certain processing [3,4,5,6,7,8,9,10,11].

This article deals with detection of people in a scene being captured of the interior space of a building. The aim of this study is to create our own laboratory solution for the detection of persons in the room with the subsequent analysis of the data applicable for the intelligent building control system [12,13,14,15,16,17]. Other approaches to dealing with the issue of person detection apply principles different from detection from the image signal. For example, References [18,19] discuss modern methods of 3D detection of persons using the N2-3DDV-Hop method. Such and other methods described in References [18,20] offer position information in 3-dimensional space. In contrast to these methods, image detection is focused on information from the projection of space into a 2D image matrix. In Reference [21], a similar problem is addressed using real hardware. However, image processing is handled differently here. The solution described in this article is intended for implementation using a built-in system hardware type. Detection should work without the use of complex algorithms, using convolutional methods [22,23] such as neural networks [24,25,26], using neural classifiers for the purpose of detecting persons as in Reference [27], or methods of detection of persons using the Haar-cascade classifier as in Reference [28], with the simplest possible subsequent implementation on built-in devices [29,30,31]. Built-in devices for detecting persons can be based on the use of image sensors, similarly as described in solutions [32,33,34], in the form of a smart camera sensor with the function of preprocessing image data into binary images with white dots indicating the position of a person in the scene, a smart camera sensor for detecting the background of the scene and foreground of the scene as the position of the found person as in Reference [35], smart camera sensor with the function of neglecting the dynamic background as in Reference [36], a smart camera sensor for Histogram of Oriented Gradients (HOG) image data processing as in Reference [37], or a specialized solution of the System on Chip (SOC) coping with basic image processing tasks such as edge detection in References [38,39], as an edge detector [40], or a solution with a low-power smart CMOS image sensor used to detect persons for indoor and outdoor use as in Reference [41]. Other image processing solutions, such as edge detection using digital parallel pulse computation [42], a non-parallel Sobel edge detector addressed by a smart camera sensor [43], discuss similar solutions that serve as sources of information providing a wide range of possible alternative solutions.

Finding the persons of the solution stated consists in finding a suspicious object in the scene, checking that the object is of a sufficient size, and checking that it may be an existing finding. This is followed by evaluation and printing of the information obtained for further possible processing, for example by the intelligent building control system [44,45,46]. Detection and localization is described in References [47,48], which are texts on camera systems for finding objects in an image matrix; in References [49,50,51], the methods of locating objects/persons in the image are discussed. References [48,52] were used as sources in tracing, and text [2] was used as a source of information on tracing using calibration methods and inverse kinematics methods as another direction in dealing with the issue of tracing.

The system devised finds suspicious objects or potential persons by subtracting two frames from each other. For example, subtraction of the current and previous frame processed, when the methods creating a static or dynamic background (replacing the previous frame found in the basic model of the system) were tested. References [38,42,53] were used as sources for work with a dynamic background and subsequent subtraction of the area of interest from the background—addressing similar solutions as this work, but in a different way. The binarized difference in brightness levels of these frames is subsequently subjected to a check of the size of the findings. The difference is obtained based on the results of the difference frame given by the frame rate (fps) used and the thresholding algorithm sensitivity. Another solution applied is the use of the Horn-Schunck method of optical flow [54,55], whose output is, similarly as the difference method output, binarized and further processed by decision algorithms, see Reference [56]. The detection, localization and tracing solutions described in this text are addressed here using only a camera sensor, comparing to other methods using a laser scanner, ultrasonic sensors, PIR sensors, as in References [1,57,58,59].

Persons are detected based on the assumed dimensions of their silhouettes. Since a person’s silhouette can take different shapes and dimensions in different camera shots (depending on the optical distortion and the location of the lens in the room), the parameters of this detection filter must be set to a particular scene. They discuss, in more detail, the basic methods of image processing for systems with low energy demands [40,60,61]. A comparison of available camera sensors for low-power systems is provided in Reference [62], but another source of image data was used in this work. In this work, a scene from the ceiling camera (Type—ASUS ZenFone 2 ZE551ML mobile phone component) located in the middle of the room using a 170${}^{{}^{\circ}}$ additional camera lens was applied. In this solution, an unpreprocessed video signal is used, that is, similar to the output of the precialized circuits mentioned in References [63,64]. The description of background detection algorithms, detection of suspicious findings and the algorithm of the final process of distinguishing the persons detected from other dynamic objects is provided in the following section.

The proposed methods in the final version of the solution are designed for low-power systems, ideally based on an SOC (System on a Chip) technology, which will be employed for the use in intelligent building applications, which is more suitable for this issue than using high-power PC graphics cards as in Reference [65]. Solutions using special chips addressing the basic tasks of image processing are provided, for example, in Reference [66], where such a system can be used for data preprocessing of a more complex system. The primary benefit of the study is the testing of the individual detection methods applied on a test video sequence including possible interfering light effects in the room. The solution proposed can be used for implementation on built-in devices.

This concept deals with the detection of persons from originally untransformed image data of an RGB camera for common use. The paper is focused on Low-Cost, Low-Power, Low Complexity–hereinafter (L-CPC) image recognition system research and it give us limitation for possible colour model. RGB colour model for algorithms and methods comparison is chosen because of image CCD and CMOS sensors common output signal. There are other necessary additional calculations for transforming to HSV, HSB, HSL colour models [67,68,69]. Of course, these models could be better for recognition results, but it is not applicable for L-CPC image recognition system with request for the highest possible process speed and the lowest computational load [70,71]. The option of use in conjunction with the aforementioned methods would be possible for these purposes of the thermal infrared camera. The error factor entered in the form of brightness deviations (caused, for example, by sharp sunlight entering the room through the window) could thus be replaced by thermal deviations in the scanned part of the scene.

Section 2 describes differential methods for detecting persons from the input video signal. Our three detection methods using computation towards a static background, three methods using computation towards a dynamic background and a method using the principle of optical flow are described here.

Section 3 describes the applied methods of adjusting the detected findings and the method of their filtering, identification, and description.

Section 4 describes the methods of evaluating the quality of detection of persons of each of the described methods. The evaluated sections of the test video used are described here, followed by the evaluation of the detection quality.

Section 5 describes the overall evaluation of the work and the results contained therein.

## 2. Methods

The developed and implemented methods described in the article were specified according to the differences in the approach to detecting the image background of the original image sequence. This not quite traditional approach based on recognition algorithms only on the variability of background detection. This way was chosen by the authors of the research work mainly with regards to own experiences and from selected literature [54,55,72,73]. Traditionally from others, the procedure for background recognition is always described, but this is not a fundamental problem and the core of the research activities. From our point of view, the variability of background detection methods is crucial for human movements recognizing. During the research, we analysed several original background detection algorithms, which were inspired by the available literature. By adapting the methods for presented detection problem, we were able to design our own original solution for each of the algorithm. The individual designed and analysed methods are described in detail below and evaluated with respect to the object recognition accuracy and success rate.

The methods of differentiating the current frame from the background can be based on both a static background and a dynamic background, see References [72,74]. Although both methods may have a plethora of variations of creation of the backgrounds used and their application in differential search for suspicious objects, there are a total of six custom solutions to this problem, three to find the difference towards the static background where each test frame is compared to a fixed frame, and three towards the dynamic background where each current test frame is compared to a background frame dynamically adjusted based on *n*-previous frames.

- The method of differentiating the current frame towards the static background, see Reference [72].
- The method of differentiating the current frame towards the dynamic background, see Reference [72].
- The method of optical flow, see Reference [55].

**Figure 1:**
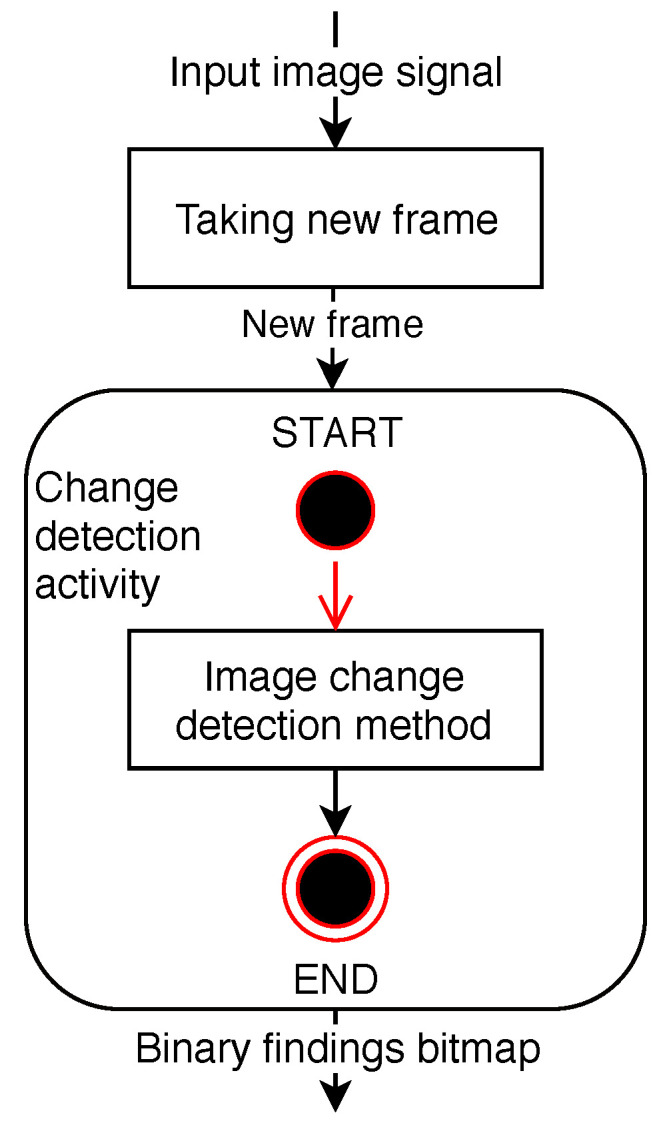
Diagram of frame change detection activity.

The changes detected in the frame are found to be appropriately thresholded and are then distributed to other parts of the program as a binary map of the objects found as described in the diagram of the activity applied in Figure 1.

Appropriate selection of the threshold for adjusting the output image results in filtering out low-contrast changes. These changes may represent slight added shadows, or large changes in brightness may also be perceived as an undesirable finding. In order to increase the robustness of the filtration system, it would be advantageous to use two threshold levels, that is, multi-level thresholding. However, in the described solution, such filtration was not used in order to demonstrate the behaviour of the described detection methods.

The selected methods are structured according to the type of work with the background image data of the detected persons. The individual versions were set up for laboratory testing, within the test video sequence, so that each of the versions achieved the highest possible success rate in detecting people. The purpose of the mutual comparison of methods very susceptible to changes in the image and sudden changes of immune methods is to point out their possible applicability in various applications. Due to the complexity of the analysis of object recognition methods, we decided to introduce as many versions and types of methods as possible. These allow embedded systems with limited computing capabilities to recognize object recognition in the image. The only complete analyses of different method versions give us complex and unquestionable answer to the most optimal algorithms.

### 2.1. The Method of Differentiating the Current Frame towards the Static Background

**Figure 2:**
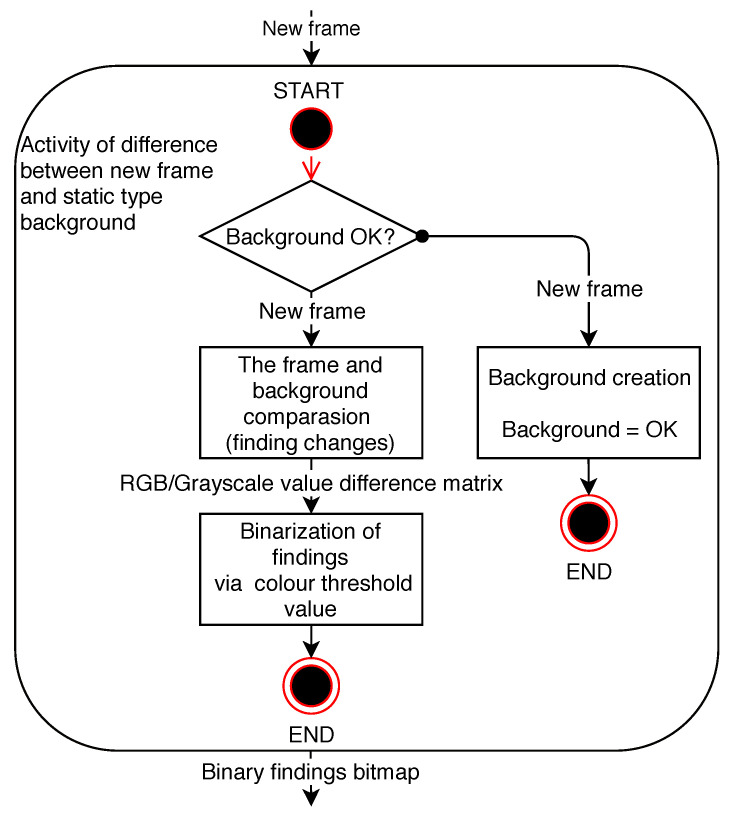
Diagram of the difference method activity towards the static background.

For clarity purposes, the method is hereinafter referred to as Method 1. This method uses the differences between the current frame and the background frame. A static background is considered to be a background fixed for the duration of the program run or for a period otherwise specified. Typically, such a background is compared to more than one frame of the scene. The principle of such a method is illustrated in the diagram in Figure 2. The advantage of using this method is a considerable saving of memory space when, after the static background is finally found, it is necessary to keep only one frame of the final background.

Within this difference method of frame change detection, three methods are applied to create a static background, where the background is:

- A single unedited reference frame (hereinafter referred to as Method 1.1).
- A weighted average of the first *n*-frames (hereinafter referred to as Method 1.2).
- A background map supplemented by invariable regions of two consecutive frames made up of *n*-frames (hereinafter referred to as Method 1.3).

#### 2.1.1. A Single Unedited Reference Frame (Method 1.1)

**Figure 3:**
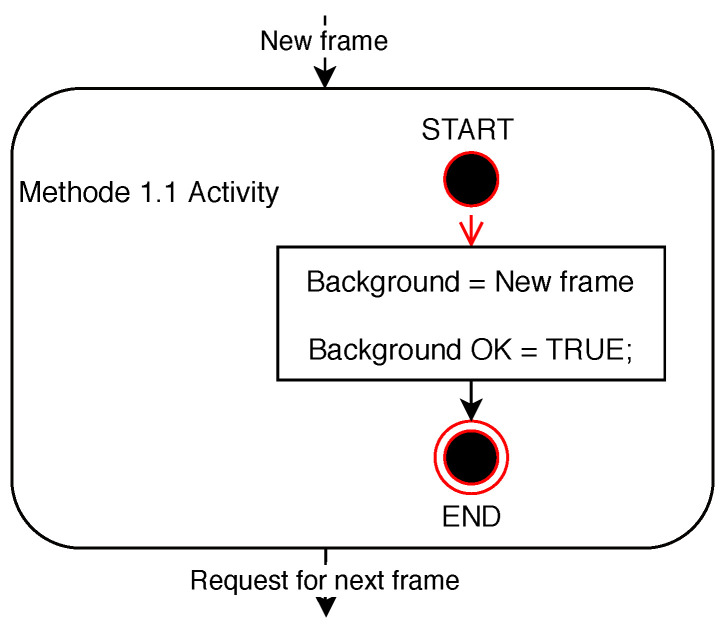
Diagram of Method 1.1 activity.

**Figure 4:**
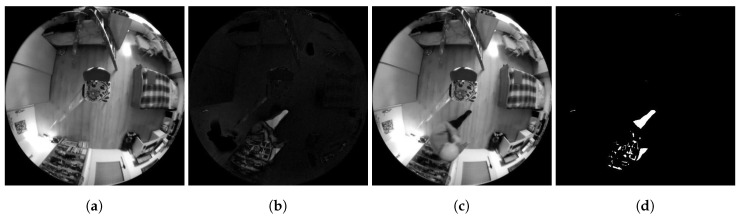
Examples of Method 1.1 functionality. (**a**) Background frame. (**b**) Difference in brightness between the currently checked frame and the background frame. (**c**) Currently checked frame. (**d**) Binarized difference in brightness between the currently checked frame and the background frame. Findings that reach a value of logical 1 (white in the frame field) are considered potential findings of a detected person.

A background created in this way is usually given by the first frame of the video sequence. The frame intended for the background should be based on the static scene in the image, ideally a scene devoid of objects dynamically moving at the time of taking the picture. Such a condition for capturing the background image ensures a minimum error rate of the subsequent detection of dynamically moving objects in future frames of the video sequence. The principle of the method is illustrated in the diagram in Figure 3. However, using this method of finding a background for change detection in the frame is very susceptible to changes in the lighting conditions in the scene of the frame compared to this background. An example of using this method is shown in a series of frames Figure 4.

#### 2.1.2. A Weighted Average of the First *n*-Frames (Method 1.2)

**Figure 5:**
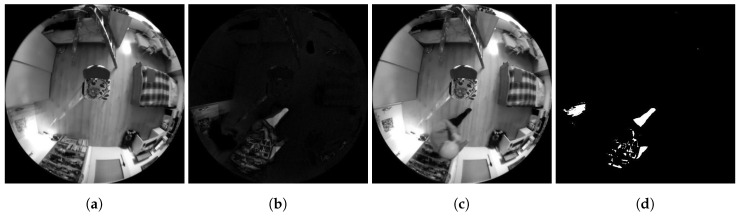
Examples of Method 1.2. functionality. (**a**) Background frame. (**b**) Difference in brightness between the currently checked frame and the background frame. (**c**) Currently checked frame. (**d**) Binarized difference in brightness between the currently checked frame and the background frame. Findings that reach a value of logical 1 (white in the frame field) are considered potential findings of a detected person.

The background thus created is based on the gradual weighted averaging of the first *n*-frames. Due to frame averaging, the effect of changing the lighting conditions in the scene is minimized, thereby minimizing the effect of unwanted changes in pixel brightness in the frames being analyzed during the subsequent detection. The purpose of this method of background creation is also neglect of possible dynamically moving objects in the scene at the beginning of the video sequence being captured. An example of using this method is shown in a series of frames Figure 5. The background can be practically created even when dynamically moving objects are present in the scene. However, such objects can introduce significant errors in the subsequent results of the detection system.

**Figure 6:**
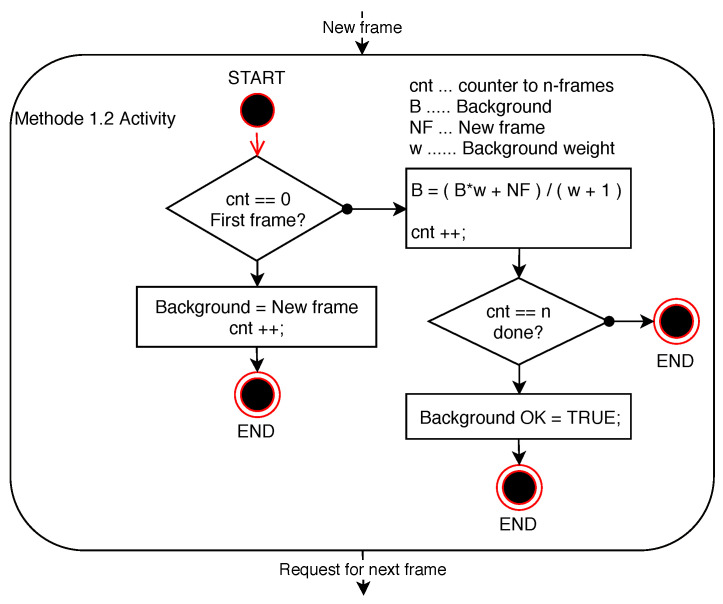
Diagram of Method 1.2 activity.

In the system code applied according to Figure 6, a background weight having a value of 5 is used. The final background is produced from the first 10 frames of the video sequence.

#### 2.1.3. A Background Map Supplemented by Invariable Regions of Two Consecutive Frames Made Up of *n*-Frames (Method 1.3)

**Figure 7:**
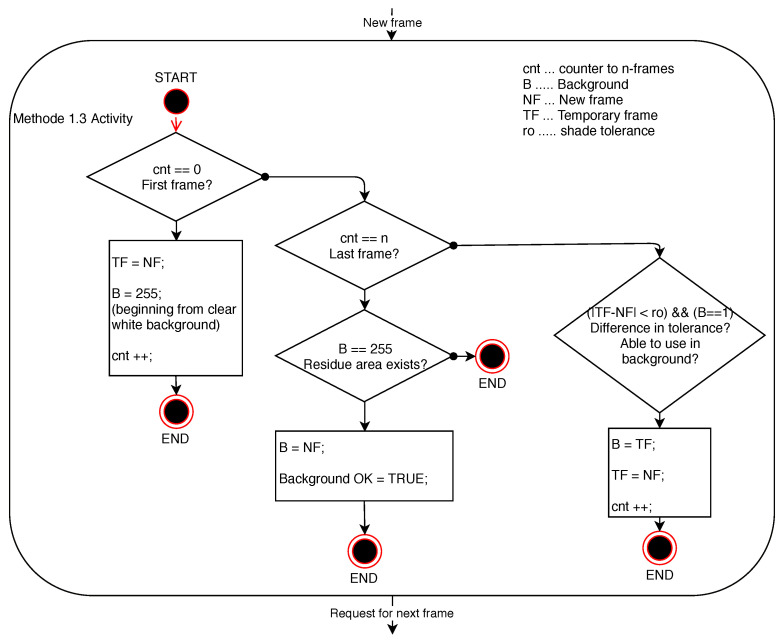
Diagram of Method 1.3 activity.

The background thus created is based on the assumption that there may be dynamically moving persons or other objects in the scene while taking pictures of the scene in order to create a static background. The principle of such a method is to gradually find fixed regions between successive frames that are gradually put together into the final background map. This algorithm is described in the diagram below in Figure 7. If this is an ideal scene that will be completely unchanged during the first two frames of the video sequence, specifically in the entire area of the frame checked, the final background will consist of pixels of the same brightness levels as the original frames. Since practically such ideal conditions cannot be achieved, there are different regions between every two frames, from tens of pixels per frame to almost the entire area of the frames compared (such an error rate may be caused by either evaluating the lighting conditions in the scene by the sensing device or by changing the lighting conditions in the scene being captured itself). Technically, by adding a static-looking region to the background map in the final background thus produced, a frame of a static-looking dynamic object may be introduced as an error in the background creation method. For example, persons sitting still in the room.

**Figure 8:**
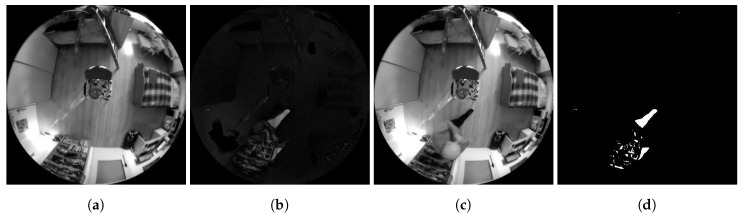
Examples of Method 1.3 functionality. (**a**) Background frame. (**b**) Difference in brightness between the currently checked frame and the background frame. (**c**) Currently checked frame. (**d**) Binarized difference in brightness between the currently checked frame and the background frame. Findings that reach a value of logical 1 (white in the frame field) are considered potential findings of a detected person.

To make the background algorithm functionality applicable also in non-ideal conditions, adjustable tolerance of the match of brightness level of shades of grey was added for the reasons of making a decision on the match of the pixel brightness value of the frames compared. An example of using this method is shown in a series of frames Figure 8.

Since, in the initial state, the background frame has a value of 255 in each pixel, that is, a brightness level corresponding to white, in the final frame of the background acquired, indeterminate regions or regions with a constant change between frames attain a brightness level of just 255. Such regions will then be evaluated as findings of objects, but of an error nature, in the subsequent binarization by thresholding.

### 2.2. The Method of Differentiating the Current Frame towards the Dynamic Background

For the sake of clarity, the method is referred to as Method 2. A background that is not compared twice to any current frame (i.e., the background is adjusted, if not changed completely, for each frame checked) is considered to be a dynamic background or, also, a variable background.

These sections describe the individual methods for the purpose of their mutual comparison. By comparing the described assumptions and the real experiment behaviour of the implemented methods, there is presented with a set of image processing options for the detection of persons by low-performance embedded camera systems used in intelligent buildings. Different methods are applied to create a various dynamic background, because it is very important for comparation of the most optimal way to background presentation. Recognition algorithm success rate for person detection directly depends on the optimal background definition. Based on these theoretical assumptions and own research experiences, we decide to analyse three methods with the most dissimilar results for background definition in our research. The chosen background detection methods are comparison frame previous to the current frame, average of all previous frames, average of *n*-previous frames. Each method brings advantages and disadvantages, which are described in text.

**Figure 9:**
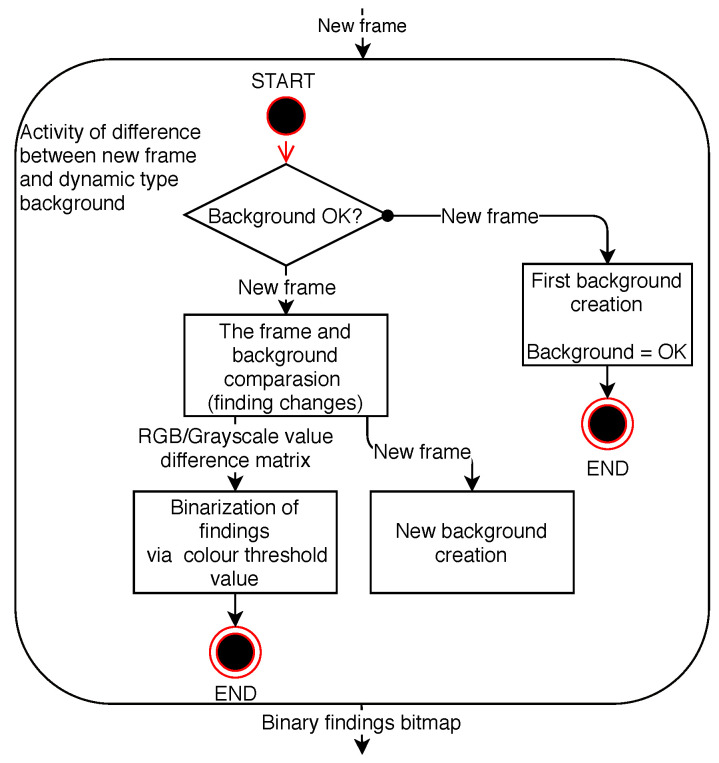
Diagram of the difference method activity of the current frame towards the dynamic background.

The difference between the current frame towards the background is calculated only after the initial background is created. A new background is created only after the current background is used for the image change detection algorithm (in the first pass of the differential part of the program, the current background is the initial background), that is, after the termination of the part of the program that uses the current background, to prevent image data collision. The new background is either a brand new frame or an existing background frame modified according to the background creation method used. Such a new background is an image function of the background of the old and new frames. The algorithm depicted above is also described in the diagram attached, in Figure 9.

Within this difference method of frame change detection, three methods are applied to create a dynamic background, where the background is:

- A frame previous to the current frame (hereinafter referred to as Method 2.1).
- Average of all previous frames (hereinafter referred to as Method 2.2).
- Average of *n*-previous frames (hereinafter referred to as Method 2.3).

#### 2.2.1. A Frame Previous to the Current Frame (Method 2.1)

**Figure 10:**
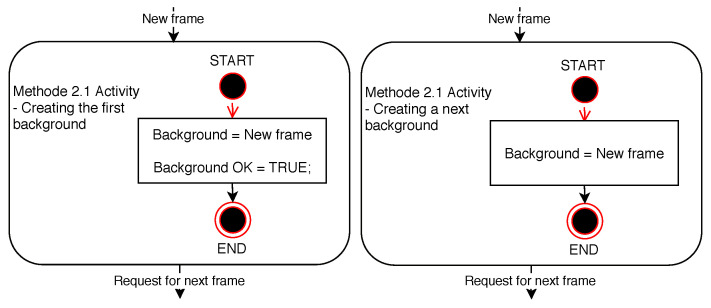
Diagrams of Method 2.1 activity.

**Figure 11:**
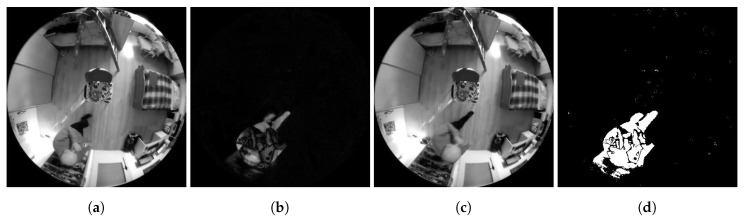
Examples of Method 2.1. functionality. (**a**) Background frame. (**b**) Difference in brightness between the currently checked frame and the background frame. (**c**) Currently checked frame. (**d**) Binarized difference in brightness between the currently checked frame and the background frame. Findings that reach a value of logical 1 (white in the frame field) are considered potential findings of a detected person.

As shown in the background creation diagrams above, this background creation method always uses the previous frame as the background for the current frame. Frame change detection based on the difference towards such a background is focused primarily on dynamically moving objects, namely objects showing a constant change between frames. The algorithm depicted is also described by the diagrams in Figure 10. The detection of an ideally constantly moving object, which can be the person being detected, is exceptionally reliable from the perspective of the theoretical assumption of the functionality of the change detection method. A great advantage of such change detection is the high resistance to changes in the lighting conditions in the scene between frames of the video sequence. The disadvantage of frame change detection, based on the principle of the method, is the subsequent increase in the error rate when the movement of the person (object) in the image scene is stopped. However, such an object, amongst the frames being dealt with, looking like a static object, cannot be found then by the detection algorithm as an object moving dynamically in the entire video sequence. The results of the behaviour of this method are shown graphically in a series of frames in Figure 11.

#### 2.2.2. Average of All Previous Frames (Method 2.2)

**Figure 12:**
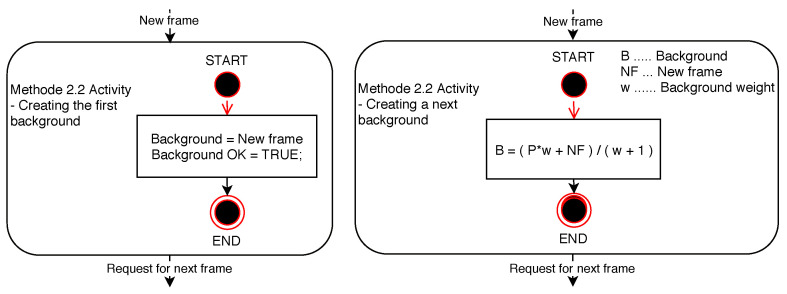
Diagrams of Method 2.2 activity.

The method of creating a dynamic background as the arithmetic mean of all the previous frames is a dynamic analogy to the above-mentioned method of creating a static background. The method is described by the diagrams in Figure 12. The background thus created is based on the gradual weighted averaging of all the frames of the video sequence. Due to frame averaging, the effect of changing the lighting conditions in the scene is minimized, thereby minimizing the effect of unwanted changes in pixel brightness in the current frames during the subsequent detection.

**Figure 13:**
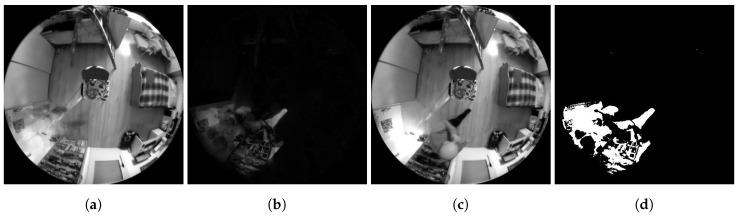
Examples of Method 2.2 functionality. (**a**) Background frame. (**b**) Difference in brightness between the currently checked frame and the background frame. (**c**) Currently checked frame. (**d**) Binarized difference in brightness between the currently checked frame and the background frame. Findings that reach a value of logical 1 (white in the frame field) are considered potential findings of a detected person.

The purpose of this method of background creation is also neglect of possible dynamically moving objects in the scene by setting a weight greater than 1, that is, the existing background weight having a value of 1 when creating a new background. By preferring the information from the previous background, neglect of the new frame is caused as well as changes in the scene associated with this neglect. Therefore, if the change in the scene is constant, it will become less important with each newly created background due to the subsequent difference of the current frame towards the background. However, the difference towards such a background is not resistant to significant sudden changes in lighting conditions in the scene. It is evident from the principle of the method that the change in the lighting conditions in the scene must be as small as possible in order to minimize errors (methods of detecting the change in the background). The weight of the previous background frame towards the current frame is a particularly important factor. The greater the weight of the previous background frame, the more negligible the errors of the newly added current frame will be in the new background. The aim of this method is to create an idealized restoring background in which the error noise caused by dynamically moving objects in the scene of the original frame is eliminated. An example of using this method is illustrated in a series of frames Figure 13.

However, the differential frame change detection in the current image from such a background is not resistant to sudden changes in the lighting conditions in the scene. Such a sudden change in lighting conditions cannot be evaluated other than an error of the method, indicating a high error rate in the entire frame.

#### 2.2.3. Average of *n*-Previous Frames (Method 2.3)

**Figure 14:**
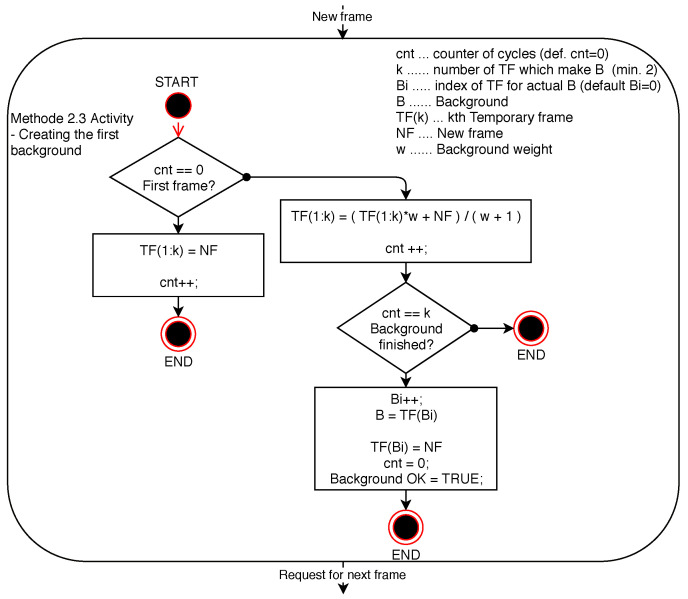
Diagram of Method 2.3 activity—creation of the initial background.

**Figure 15:**
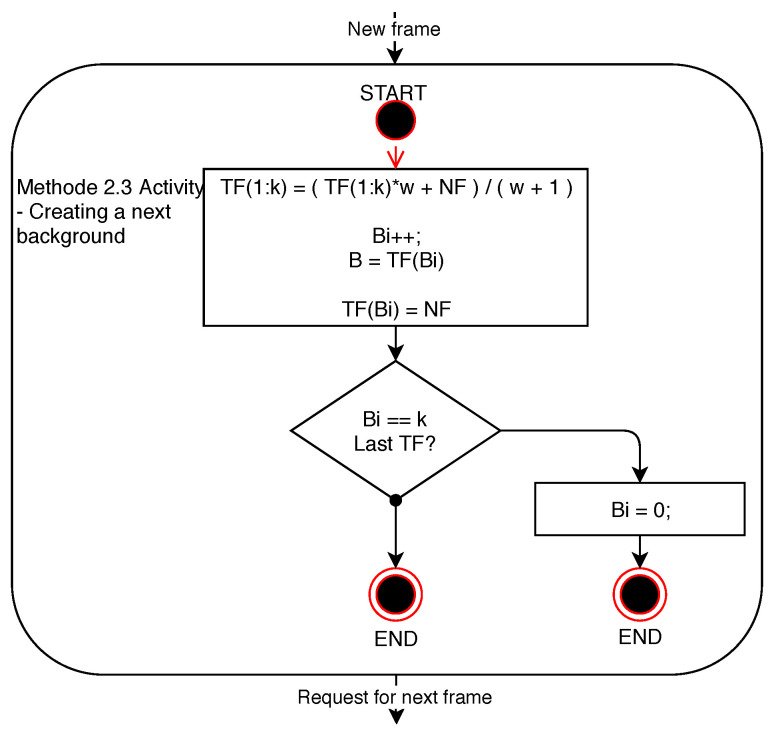
Diagram of Method 2.3 activity—creation of a new background.

Similarly to the method of difference towards the dynamic background created by averaging of all the previous frames, the functionality of the method using a background created by averaging *n*-previous frames is dependent on setting the weighting parameter of the previous frame. In addition to this adjustable parameter, it is also necessary to determine the number of frames that will form the dynamic background of the current frame when the difference is detected in the image. The weight parameter of the previous background frame, when creating a new background, affects the gradual disappearance of images of dynamically moving objects or persons in the scene in order to achieve the most accurate background for the subsequent differential detection of changes in the current frame towards the background frame. The method is described by the diagrams shown in Figure 14, describing the creation of the initial background, and Figure 15 describing the creation of each new background.

**Figure 16:**
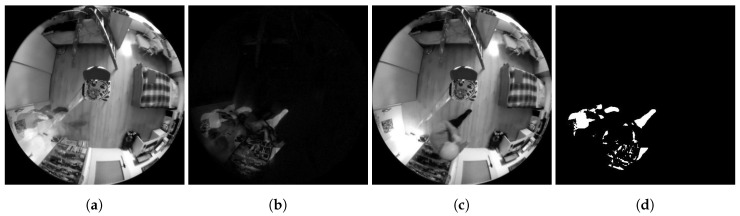
Examples of Method 2.3 functionality. (**a**) Background frame. (**b**) Difference in brightness between the currently checked frame and the background frame. (**c**) Currently checked frame. (**d**) Binarized difference in brightness between the currently checked frame and the background frame. Findings that reach a value of logical 1 (white in the frame field) are considered potential findings of a detected person.

This method is designed to eliminate the effects of dynamic object images in the scene of the current frame involved in creating a new background frame. In contrast to the aforementioned method of averaging of all the previous frames of the current frame, a limited number of previous frames are used for the current dynamic background of the current frame. This prevents the past error from being brought into the current background. The weighting calculation setting is also adjusted so that the oldest background frame took the highest weight, thus bringing the highest error rate to the resulting frame. In this way, the error detection of dynamically moving objects in the scene, whose position dynamic change is expected in the scene of the current frame, is limited. Figure 16 shows an example of the output of this method.

Of the above-mentioned methods, this method is the most memory-intensive method, because it is necessary to preserve just *n*-frames of the temporary background for the background created from *n*-previous frames (background frames are gradually completed according to the algorithms described above).

### 2.3. The Method of Optical Flow (Method 3)

**Figure 17:**
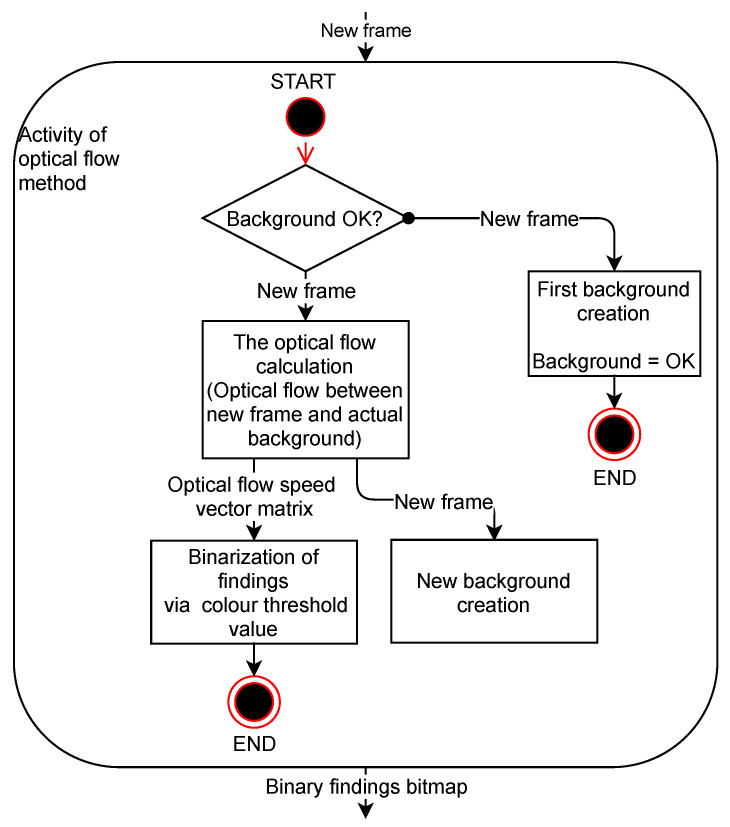
Diagram of optical flow method activity (Method 3).

For the sake of clarity, the method is referred to as Method 3. This is a method developed to detect dynamically moving objects in a scene. The program calculates a velocity vector that reflects the direction and magnitude of the velocity of motion in the image using the optical flow method and the resulting brightness function calculation. However, from this velocity vector, information about the velocity vector magnitude at each point of the image examined is taken for further processing. This information of potentially moving objects is then thresholded in order to obtain binary images of the findings. The application of the optical flow method is described in the diagram below in Figure 17.

**Figure 18:**
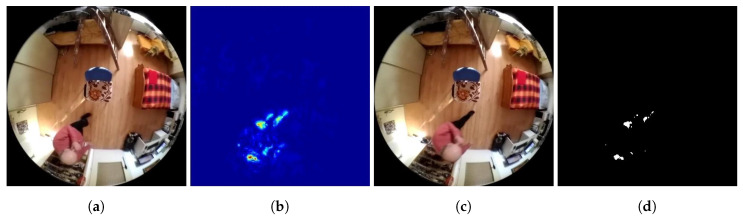
Examples of Method 3. functionality. (**a**) Background frame. (**b**) Map showing the velocity vector magnitude between the frames (dark blue = low velocity, red = high velocity) (**c**) Currently checked frame. (**d**) Binarized map of velocity vector magnitude. Findings that reach a value of logical 1 (white in the frame field) are considered potential findings of a detected person.

This is a method that is very sensitive to changes in the lighting conditions in the scene of the input frames. In contrast to the difference methods, which deal with the change in the image consisting of greyscale input frames, the method used deals with finding the change between the input frames, the current frame, and the background frame, in the original RGB colour model. Since a third-party code is used, the algorithm of this method will not be described in detail in this work. The Horn-Schunck optical flow solution algorithm is available on the MathWorks MATLAB support site [54,55,73]. An example of applying the method described to a moving object detection algorithm is shown in a series of frames in Figure 18.

## 3. Adjustment of the Objects Found

This part of adjustment of the block of the objects found is designed to adjust the dimensions and integrity of the objects found. This adjustment is achieved by means of the following steps:

1. Filter erosion of the objects found to remove image noise findings.
2. Dilatation of the objects found.
3. Erosion of the object found.

**Figure 19:**
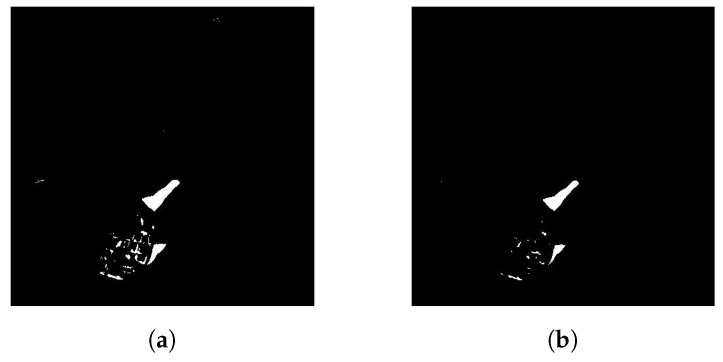
Example of adjustment of objects found in the binary image by erosion filtering. Erosion filtering of objects by 3 pixels. (**a**) The original binary image of the findings after binarization of the difference finding. (**b**) Image (**a**) filtered by noise objects using erosion.

Each of these steps has its reason in the final shape and size of the object found. Filtering the findings caused by noise in the image using erosion is a method of minimizing the potential for increasing error rates in the subsequent dilatation step of the objects found. The work uses erosion by three pixels for this purpose, as shown in Figure 19, that is, each of the objects found is reduced line by line, always by one pixel of a positive finding.

**Figure 20:**
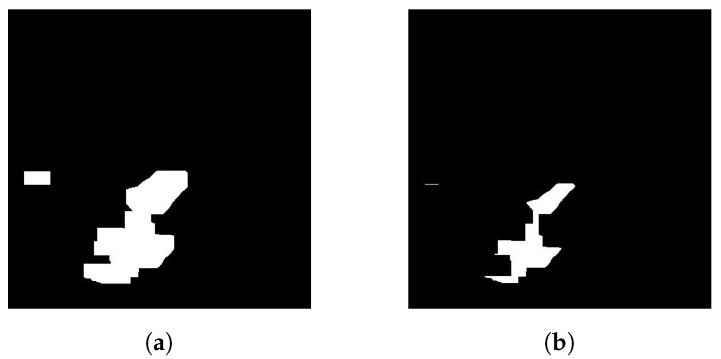
Example of adjustment of objects found in the binary image by dilatation and reverse erosion of objects by a rectangular area of 20 × 20 pixels, both in the horizontal and vertical directions. (**a**) The result of the filtered finding dilatation. (**b**) The result of the reverse erosion of the binary image (**a**).

During dilatation, the size of each object finding is extended by a user-adjustable factor of the region around the particular finding point. This step is used to combine discontinuous object findings into an undivided object. The frame dilatation effect in Figure 19b is shown in Figure 20a. However, the adjustment of the aforementioned dilatation region factor is different for the resulting binary image of each of the methods of frame change detection used, as needed. For example, when using the change detection method by means of optical flow, a significant expansion of the findings is necessary, since the findings belonging to the location of the person in the scene of the current frame may occur separately from the findings of objects belonging to the same person in the scene.

If added shadows occur, the success rate of the methods selected for the correct detection of persons in the image is often reduced. In the case of differential methods, such as Method 1.1, Method 1.2, Method 1.3, Method 2.1, Method 2.2, Method 2.3, the shadow could be perceived as a new detected person. To evaluate a sufficient change in the image, sufficient to evaluate a new person, it is thus crucial to set a threshold level for filtering the input data. To clarify this possible phenomenon, a text was added to the article (Section 2) explaining the behaviour of the described methods in the case of the occurrence of excessive shadows. In addition, the practical implementation envisages the addition of an algorithm comprising definition of areas where newcomers can appear and where they can leave the room. The article no longer deals with this functional superstructure, but it will significantly contribute to the elimination of the above-mentioned phenomenon.

### 3.1. Identification of Object Findings

The binary image of the objects adjusted is then subjected to the identification of the individual object findings.

In the first stage of identification, the positive pixels (belonging to the potential person) of each continuous pixel cluster according to the 4-neighbour rule are grouped into regions identified by the original identifier.

**Figure 21:**
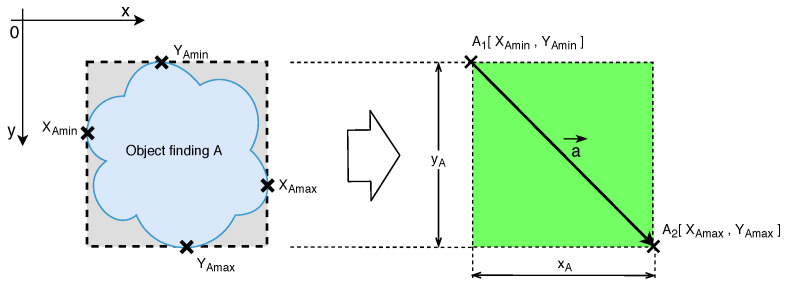
Illustration describing the marking of the object found by a rectangular marking field.

The next stage of the identification of the object found is marking into the delimited regions and their possible joining into larger units. The identified object regions found are further distributed by the vector description of the rectangular areas, that is, the description of two pixels in the image. The first, initial, point is given by the smallest horizontal pixel coordinate and the smallest vertical pixel coordinate of the object described. The second point is then given by the largest horizontal pixel coordinate and the largest vertical pixel coordinate of the object described. The principle of marking the object found is explained in the attached illustration in Figure 21.

**Figure 22:**
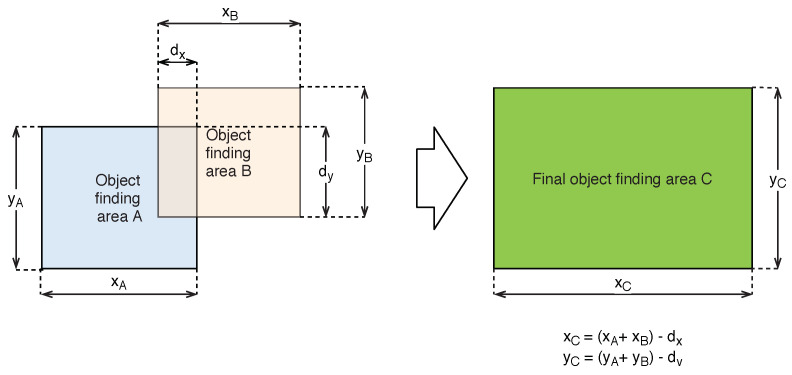
Illustrations describing the unification of the intersecting objects found into the final object.

The objects found that are thus delimited by the rectangular areas are then checked for intersection with another delimited region in the delimitation area. If they have intersection areas, they are then unified into a common region of the object, as shown in the illustration in Figure 22.

The information about the object found is inserted in a two-dimensional description field of the objects found in the frame currently checked for the reasons of further analysis. After the check and unification of the objects into marking regions are completed, the applied image is no longer used by the algorithm, as other parts of the algorithm only work with the so-called rectangular area marking vectors.

### 3.2. Filtration of Error Findings from Findings of Persons

The object findings adjusted and identified are filtered according to the dimensions of the custom rectangular delimitation and the actual pixel area of the positive pixels in the marking field.

**Figure 23:**
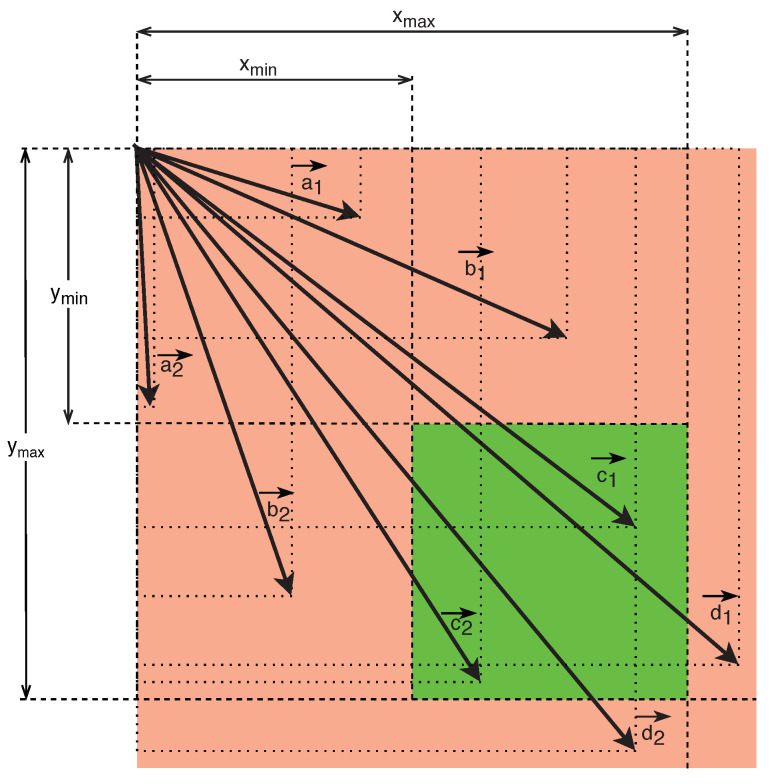
Illustration of filtering of the objects found according to the dimensions of their rectangular delimitations described by a descriptive vector.

In the above-mentioned illustration in Figure 23, vectors representing regions indicating object findings are plotted. Vectors denoted as $\vec{a_{1}}$ and $\vec{a_{2}}$ represent regions smaller than the minimum dimensions of $x_{\min}$ and $y_{\min}$, specified by the condition for filtering object findings from findings of persons. Vectors denoted as $\vec{b_{1}}$ and $\vec{b_{2}}$ represent regions, one dimension of which is less than the minimum dimension limit of $x_{\min}$ or $y_{\min}$, specified by the condition for filtering object findings from findings of persons. Vectors denoted as $\vec{d_{1}}$ and $\vec{d_{2}}$ represent regions, at least one dimension of which is more than the maximum dimension limit of $x_{\max}$ or $y_{\max}$, specified by the condition for filtering object findings from findings of persons. Thus, the findings delimited by the regions represented by vectors denoted as $\vec{a_{1}}$, $\vec{a_{2}}$, $\vec{b_{1}}$, $\vec{b_{2}}$, $\vec{d_{1}}$, and $\vec{d_{2}}$ do not meet the requirements of the filter criteria set according to the dimensions and are, therefore, not further processed as findings of persons. In contrast, vectors denoted as $\vec{c_{1}}$ and $\vec{c_{2}}$ represent regions acquiring both dimensions greater than the minimum dimensions of $x_{\min}$ and $y_{\min}$ and smaller than the dimensions of $x_{\max}$ or $y_{\max}$, specified by the condition for filtering object findings from findings of persons. Such object findings are further analyzed as findings of persons.

### 3.3. Cataloguing of Persons Found

In the block of cataloguing, the persons found, represented by a description of their areas of location and size, are identified, and subsequently monitored to see if these findings are still current in the scene. It is in this part of the system for the detection of persons in intelligent buildings that the findings of persons from current frames are set in context with the findings of persons from the previous frames. The cataloguing block works with a two-dimensional array of information, in which each current finding is assigned with one row of this array of information. The array of information is the bearer of information about the persons currently found as well as error findings in the scene, which are assigned with a lifespan value of each of the findings within this block.

**Figure 24:**
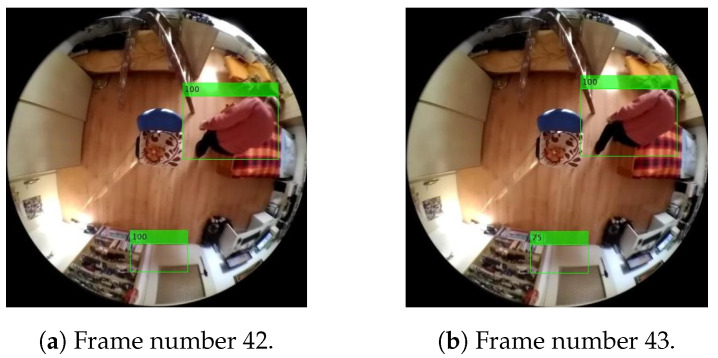
Example of reducing the lifespan at an error finding. The illustration shows the real results of Method 1.1, with a lifespan of 1s, i.e., 4 frames at a frame rate of 4fps.

The index that is assigned to each person newly found or a person currently restored at the maximum possible user-selectable value, called the “maximum lifespan of a person finding”, is designated as the lifespan of person findings as shown in the frame in Figure 24a. Each time the program loop passes through, the lifespan value of each person found is decremented as shown in the frame in Figure 24b. If the person finding in the array of current findings is no longer restored during the period of his/her non-zero lifespan and the person finding has a lifespan of zero, the person finding is removed from the array of current findings as an outdated or error finding.

**Figure 25:**
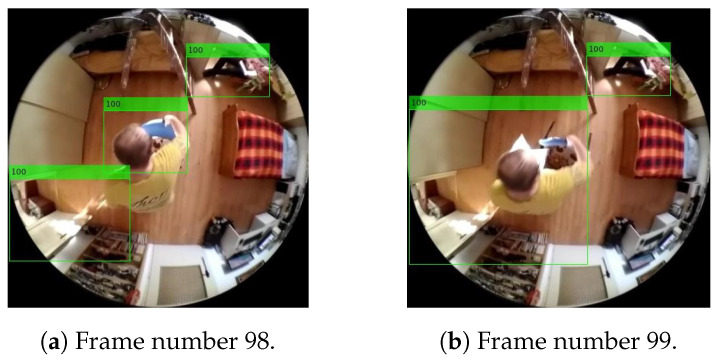
Example of merging person findings. The illustration shows the real results of Method 1.1, with a lifespan of 1s, i.e., 4 frames at a frame rate of 4fps.

As part of its identification, each of the new person findings is compared to the person findings listed in the array of information about the currently occurring person findings in the scene. The evaluation of conformity of the person findings is conducted by means of detecting the intersections of the mutually checked rectangular areas of the delimitations of the findings. The effect of detecting the intersections of the areas found is shown in the frames in Figure 25. In the case of finding the intersection of the areas checked, the existing finding is overwritten by the new finding and supplemented with the maximum lifespan of the person finding.

In the example below, illustrating the structure of the individual output data of the final system, the information characterizing the two objects from Figure 24 is shown, namely Figure 24b, when one of the objects/potential-persons found acquires a 100% lifespan, wherein the lifespan of the other one is already reduced compared to the frame in Figure 24a, where both findings have a 100% lifespan.

Table 1 provides an example of all current person findings, in the frame in Figure 24b, including their current lifespan. Since, when producing these examples, the maximum, that is, 100% lifespan, is set to 4, that is, 4 frames, the most current person finding, mentioned above in the tables of the initial findings and unified person findings, is correctly evaluated by the lifespan index in the first row. The next finding, having a lifespan index value of 3, is the finding that was last updated during the previous program pass, that is, in the previous input frame. Furthermore, the table of output information transferred to the superior system includes data on: the number of custom pixels belonging to the person found and information on location of the individual findings in the matrix of the image checked.

## 4. Results

For each of the methods used, the person detection system was applied to the same video sequence, which was designed to simulate the normal operating conditions of a camera in intelligent buildings. The test video was divided into 12 partial event sections, which were individually evaluated and statistically processed.

Table 2 depicting the test video event sections describes the activities of the persons in each section. These test video sections are designed to capture the most common possible behaviour of persons in a scene being captured by the camera system of an intelligent building. It includes entrance, exit, movement of persons, occasional stopping of persons and shifting of objects in the scene.

### 4.1. Verification of the Results

In order to determine the correct function of the algorithms applied, it was necessary to create a verification template of the results. The results of the verification had to be generated for each of the frames tested so as to sufficiently assess the quality of the detection of the persons. In the test video sequence, the verification of the results achieved was thus conducted on the frame-by-frame basis.

The final weighted detection quality assessment (K) is a result of the sum of the individual weights of the quality criteria (*K*) multiplied by a percentage of the quality criteria met $p_{K}$. The weights of the quality criteria are determined by the Saaty multi-criteria evaluation method according to the source cited below.

The values of the criteria weights are determined using geometric means of the rows of the Saaty matrix, plotted in the Table 3. If these row geometric means are standardized, the standardized weights of the set of criteria being dealt with are obtained [15].

(1) $$v_{i}=\frac{G_{i}}{\sum_{i=1}^{n}G_{i}},$$

where $v_{i}$ is the standardized weight of the i-th criterion, $G_{i}$ is the geometric mean of the i-th criterion, and *n* is the total number of criteria.

The work uses five quality criteria. Each of these criteria stated expresses the correctness of person findings detected in the scene being captured.

- $K_{1}$ Criterion: Average percentage of custom pixels of person findings in the field for marking persons
- $K_{2}$ Criterion: Average lifespan of person findings
- $K_{3}$ Criterion: Average percentage intersection of the areas of marking of the person finding with the verification pattern
- $K_{4}$ Criterion: Average error of location of marking of person findings with the intersection in the verification pattern
- $K_{5}$ Criterion: Success rate of the detection of correct person findings in relation to all current findings.

The values provided in the rows and columns of Table 3 are based on the importance of one criterion in relation to another. There are values of 1 in the diagonal because criterion $K_{1}$ in the row is equivalent to criterion $K_{1}$ in the column. The highest criterion is a value of 9, wherein the criterion acquiring the value of 9 is absolutely more significant than the criterion in the relevant column.

The preferences between the individual criteria are determined expertly according to the requirement for strictness of the final weighted detection quality assessment.

Since it is necessary to include the information on whether or not the quality assessment criteria are met in such a system of weights devised in order to conduct a cumulative assessment of the system detection quality results, the resulting weighted quality assessment is obtained by the following calculation:

(2) $$K=\sum_{i=1}^{n}\left(v_{i}\cdot \frac{p_{k_{i}}}{100}\right),$$

where the weighted detection quality assessment is denoted as K, $v_{i}$ is the standardized weight of the i-th criterion, and $p_{k_{i}}$ is the percentage of the i-th criterion.

By using $p_{k_{i}}$ as a percentage of meeting the i-th criterion, the resulting value of the weighted quality assessment K is in the range of 0 to 1. The weighted detection quality assessment K equals zero if each of the percentages of the i-th criterion equals zero, or the person finding detected is negative, or, also, ideally incorrect. Conversely, K equals 1 if the ideal person finding detected is correct. As it is obvious from the following relationships:

(3) $$K_{\max}=\sum_{i=1}^{n}\left(v_{i}\cdot \frac{p_{k_{i}}\max}{100}\right)=1,$$

(4) $$K_{\min}=\sum_{i=1}^{n}\left(v_{i}\cdot \frac{p_{k_{i}}\min}{100}\right)=0.$$

In practice, however, the ideal state where K equals 1 is very difficult to achieve. This is because the percentage of meeting the i-th criterion, $p_{k_{i}}$, is below 100% of the ideal state for each person detection quality assessment criterion.

**Figure 26:**
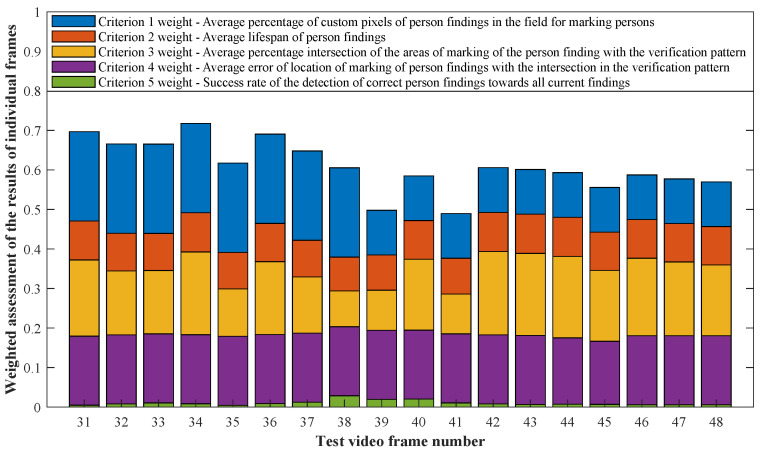
Distribution of the individual criterion weights in the practical result of the video section.

The example shown in the figure (Figure 26) indicates the distribution and importance of the individual weights of the weighted detection quality rating to the final rating (K). Table 4 is shown as an example of the summary results of the selected method (Method 1.1) in the individual sections, including the average measurement error.

The following graphs of the results of Methods 1.1, 1.2, 1.3, 2.1, 2.2, 2.3, 3 and the results of each of them when changing the lifespan parameter of the persons found show that the optimal value of the lifespan parameter of the persons found plays a significant role for each of the detection algorithm configurations applied.

**Figure 27:**
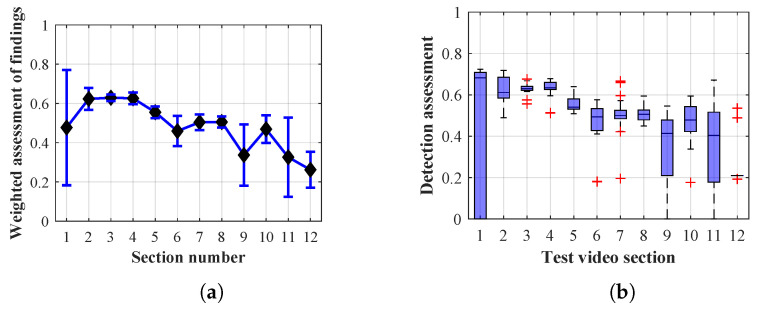
Results of Method 1.1 with a lifespan of 1 frame. (**a**) the weighted average rating with the average mean absolute error of the weighted rating and (**b**) boxplots of the distribution of the statistical sets of the results of the individual sections evaluated indicating: minimum, 1st quartile, median, 3rd quartile, maximum.

**Figure 28:**
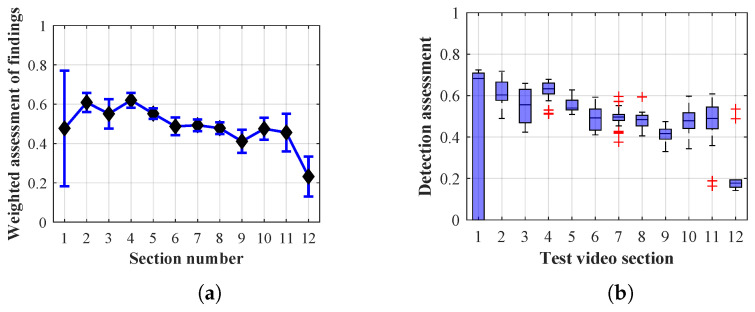
Results of Method 1.1 with a lifespan of 12 frames. (**a**) the weighted average rating with the average mean absolute error of the weighted rating and (**b**) boxplots of the distribution of the statistical sets of the results of the individual sections evaluated indicating: minimum, 1st quartile, median, 3rd quartile, maximum.

**Figure 29:**
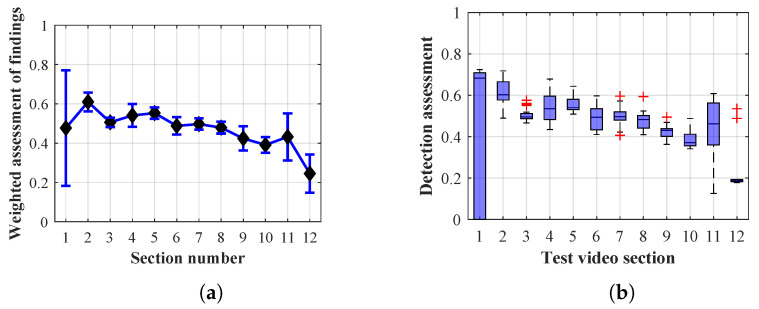
Results of Method 1.1 with a lifespan of 40 frames. (**a**) the weighted average rating with the average mean absolute error of the weighted rating and (**b**) boxplots of the distribution of the statistical sets of the results of the individual sections evaluated indicating: minimum, 1st quartile, median, 3rd quartile, maximum.

As it is obvious from the example of the results of the average weighted rating of Method 1.1 shown in Table 4, this functional configuration achieved the best rating with a lifespan of the person found of 12 frames (i.e., a lifespan of 3 s at 4 fps), with an average measurement error of 7.5%. The results shown in Table 4 are indicated in the attached figures (Figure 27a, Figure 28b and Figure 29a).

Many methods, as illustrated in the resulting graphs, from Figure 27, Figure 28, Figure 29, Figure 30, Figure 31, Figure 32, Figure 33, Figure 34, Figure 35, Figure 36, Figure 37, Figure 38, Figure 39, Figure 40, Figure 41, Figure 42, Figure 43, Figure 44, Figure 45, Figure 46 and Figure 47, had a significantly increased error rate in the first and last section. This is caused by the inconsistent presence of detectable persons in the room being checked and their erroneous assessment, either due to the background creation delay or the delay caused by the excessively long lifespan of the persons detected.

#### 4.1.1. Evaluation of Method 1.1

By comparing the results of Method 1.1, evaluation at a lifespan of 1, 12, and 40 frames, it is ascertained that the best detection results were achieved within video sections 2 to 5. A graphical representation of the evaluation of the configurations used is shown, for results with a lifespan of 1 frame, in Figure 27, for results with a lifespan of 12 frames, in Figure 28, for results with a lifespan of 40 frames in Figure 29. In subfigure (b), the selected figures show boxplots of the statistical distribution of the weighted evaluation in selected sections into quartiles. The final summary of the results of all the methods in Table 5 includes the weighted average evaluation results for all the sections of the video, wherein the best weighted and scaled scores were achieved with Method 1.2 having a lifespan of 12 frames.

#### 4.1.2. Evaluation of Method 1.2

**Figure 30:**
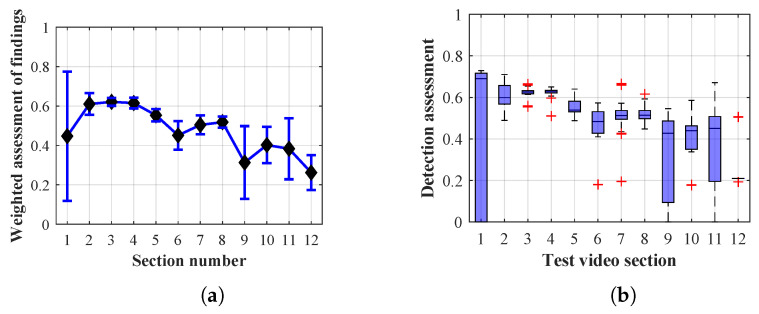
Results of Method 1.2 with a lifespan of 1 frame. (**a**) the weighted average rating with the average mean absolute error of the weighted rating and (**b**) boxplots of the distribution of the statistical sets of the results of the individual sections evaluated indicating: minimum, 1st quartile, median, 3rd quartile, maximum.

**Figure 31:**
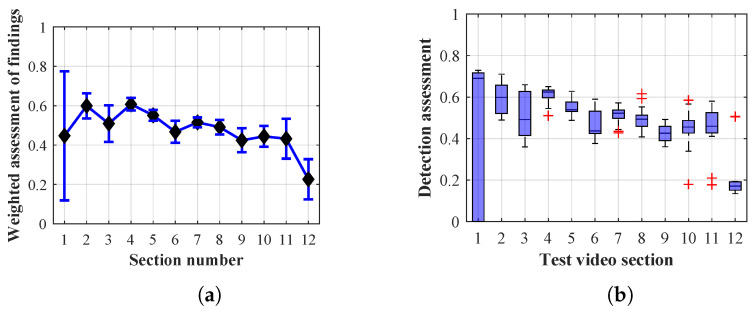
Results of Method 1.2 with a lifespan of 12 frames. (**a**) the weighted average rating with the average mean absolute error of the weighted rating and (**b**) boxplots of the distribution of the statistical sets of the results of the individual sections evaluated indicating: minimum, 1st quartile, median, 3rd quartile, maximum.

**Figure 32:**
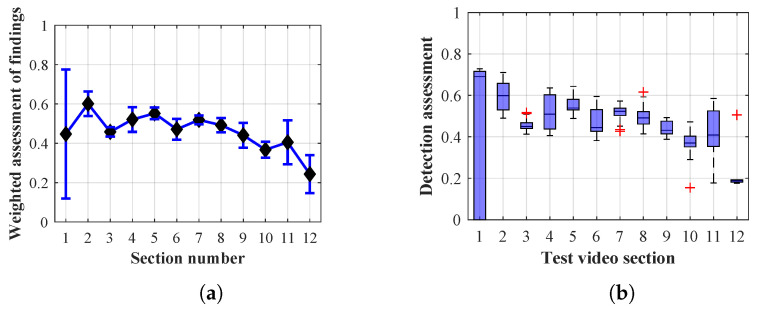
Results of Method 1.2 with a lifespan of 40 frames. (**a**) the weighted average rating with the average mean absolute error of the weighted rating and (**b**) boxplots of the distribution of the statistical sets of the results of the individual sections evaluated indicating: minimum, 1st quartile, median, 3rd quartile, maximum.

By comparing the results of Method 1.2, evaluation at a lifespan of 1, 12, and 40 frames, it is ascertained that the best detection results were achieved within video sections 2 to 4. A graphical representation of the evaluation of the configurations used is shown, for results with a lifespan of 1 frame, in Figure 30, for results with a lifespan of 12 frames, in Figure 31, for results with a lifespan of 40 frames in Figure 32. In subfigure (b), the selected figures show boxplots of the statistical distribution of the weighted evaluation in selected sections into quartiles. The final summary of the results of all the methods in Table 5 includes the weighted average evaluation results for all the sections of the video, wherein the best weighted and scaled scores were achieved with Method 1.2 having a lifespan of 12 frames.

#### 4.1.3. Evaluation of Method 1.3

**Figure 33:**
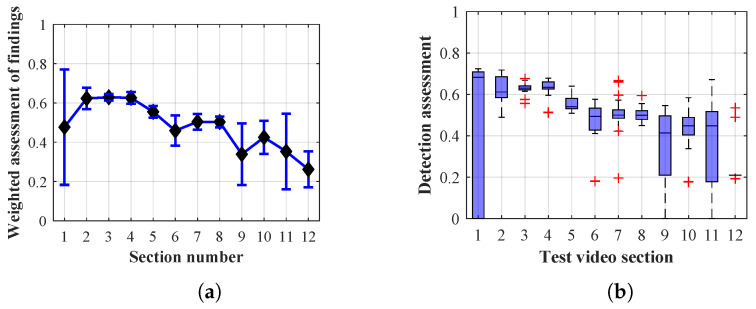
Results of Method 1.3 with a lifespan of 1 frames. (**a**) the weighted average rating with the average mean absolute error of the weighted rating and (**b**) boxplots of the distribution of the statistical sets of the results of the individual sections evaluated indicating: minimum, 1st quartile, median, 3rd quartile, maximum.

**Figure 34:**
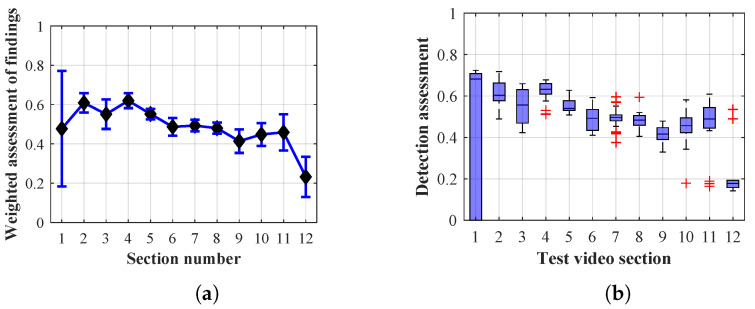
Results of Method 1.3 with a lifespan of 12 frames. (**a**) the weighted average rating with the average mean absolute error of the weighted rating and (**b**) boxplots of the distribution of the statistical sets of the results of the individual sections evaluated indicating: minimum, 1st quartile, median, 3rd quartile, maximum.

**Figure 35:**
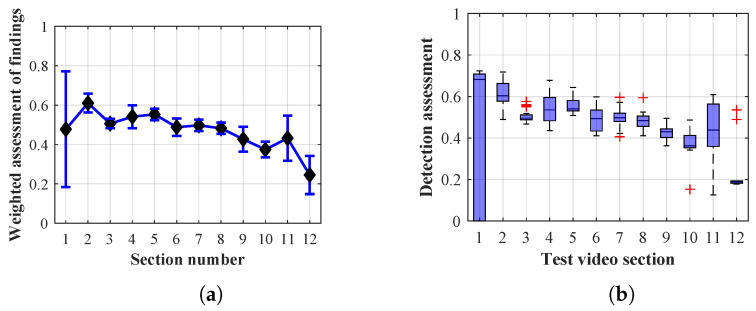
Results of Method 1.3 with a lifespan of 40 frames. (**a**) the weighted average rating with the average mean absolute error of the weighted rating and (**b**) boxplots of the distribution of the statistical sets of the results of the individual sections evaluated indicating: minimum, 1st quartile, median, 3rd quartile, maximum.

By comparing the results of Method 1.3, evaluation at a lifespan of 1, 12, and 40 frames, it is ascertained that the best detection results were achieved within video sections 2 to 4. A graphical representation of the evaluation of the configurations used is shown, for results with a lifespan of 1 frame, in Figure 33, for results with a lifespan of 12 frames, in Figure 34, for results with a lifespan of 40 frames in Figure 35. In subfigure (b), the selected figures show boxplots of the statistical distribution of the weighted evaluation in selected sections into quartiles. The final summary of the results of all the methods in Table 5 includes the weighted average evaluation results for all the sections of the video, wherein the best weighted scores were achieved with Method 1.3 having a lifespan of 12 frames; the best scaled scores were achieved with a lifespan of 40 frames.

#### 4.1.4. Evaluation of Method 2.1

**Figure 36:**
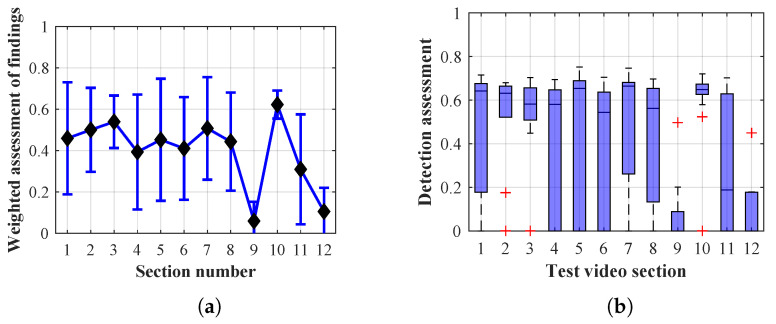
Results of Method 2.1 with a lifespan of 1 frames. (**a**) the weighted average rating with the average mean absolute error of the weighted rating and (**b**) boxplots of the distribution of the statistical sets of the results of the individual sections evaluated indicating: minimum, 1st quartile, median, 3rd quartile, maximum.

**Figure 37:**
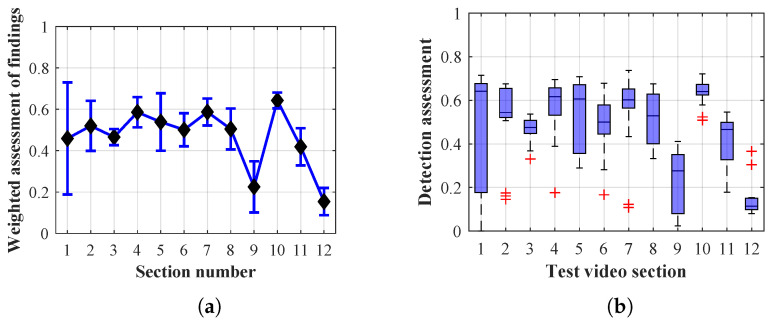
Results of Method 2.1 with a lifespan of 12 frames. (**a**) the weighted average rating with the average mean absolute error of the weighted rating and (**b**) boxplots of the distribution of the statistical sets of the results of the individual sections evaluated indicating: minimum, 1st quartile, median, 3rd quartile, maximum.

**Figure 38:**
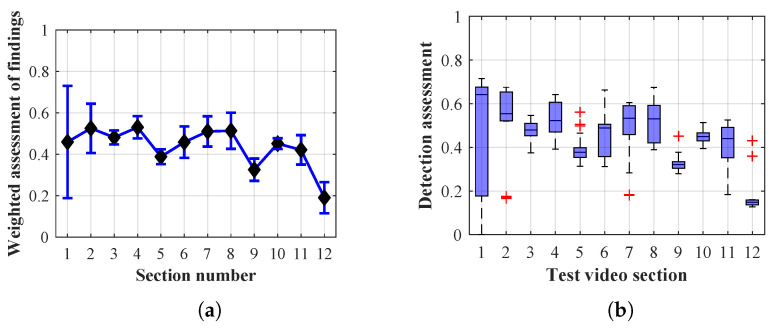
Results of Method 2.1 with a lifespan of 40 frames. (**a**) the weighted average rating with the average mean absolute error of the weighted rating and (**b**) boxplots of the distribution of the statistical sets of the results of the individual sections evaluated indicating: minimum, 1st quartile, median, 3rd quartile, maximum.

By comparing the results of Method 2.1, evaluation at a lifespan of 1, 12, and 40 frames, it is ascertained that, within the lifespan increase, an increase in the stability of the results of the weighted detection assessment in the individual sections was achieved. A graphical representation of the evaluation of the configurations used is shown, for results with a lifespan of 1 frame, in Figure 36, for results with a lifespan of 12 frames, in Figure 37, for results with a lifespan of 40 frames in Figure 38. In subfigure (b), the selected figures show boxplots of the statistical distribution of the weighted evaluation in selected sections into quartiles. The final summary of the results of all the methods in Table 5 includes the weighted average evaluation results for all the sections of the video, wherein the best weighted scores were achieved with Method 2.1 having a lifespan of 12 frames; the best scaled scores were achieved with a lifespan of 40 frames. As it is obvious from the results in both Figure 36 and Table 5, Method 2.1 achieves very poor results with a lifespan of findings lasting 1 frame.

#### 4.1.5. Evaluation of Method 2.2

**Figure 39:**
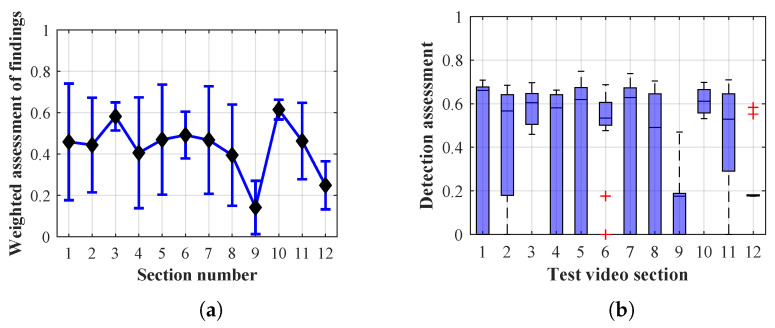
Results of Method 2.2 with a lifespan of 1 frames. (**a**) the weighted average rating with the average mean absolute error of the weighted rating and (**b**) boxplots of the distribution of the statistical sets of the results of the individual sections evaluated indicating: minimum, 1st quartile, median, 3rd quartile, maximum.

**Figure 40:**
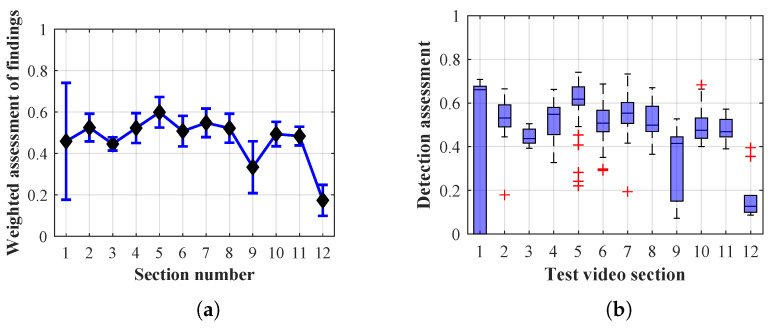
Results of Method 2.2 with a lifespan of 12 frames. (**a**) the weighted average rating with the average mean absolute error of the weighted rating and (**b**) boxplots of the distribution of the statistical sets of the results of the individual sections evaluated indicating: minimum, 1st quartile, median, 3rd quartile, maximum.

**Figure 41:**
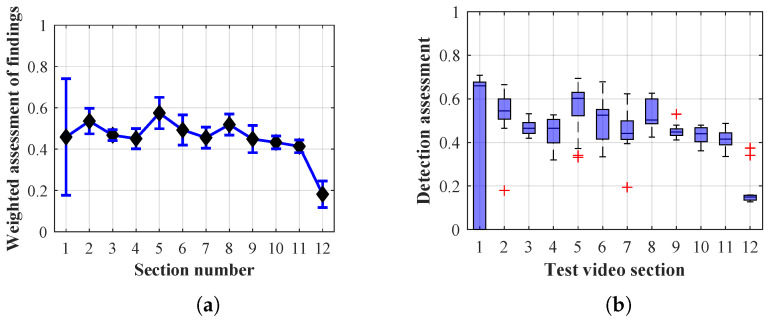
Results of Method 2.2 with a lifespan of 40 frames. (**a**) the weighted average rating with the average mean absolute error of the weighted rating and (**b**) boxplots of the distribution of the statistical sets of the results of the individual sections evaluated indicating: minimum, 1st quartile, median, 3rd quartile, maximum.

By comparing the results of Method 2.2, evaluation at a lifespan of 1, 12, and 40 frames, it is ascertained that, within the lifespan increase, an increase in the stability of the results of the weighted detection assessment in the individual sections was achieved. A graphical representation of the evaluation of the configurations used is shown, for results with a lifespan of 1 frame, in Figure 39, for results with a lifespan of 12 frames, in Figure 40, for results with a lifespan of 40 frames in Figure 41. In subfigure (b), the selected figures show boxplots of the statistical distribution of the weighted evaluation in selected sections into quartiles. The final summary of the results of all the methods in Table 5 includes the weighted average evaluation results for all the sections of the video, wherein the best weighted scores were achieved with Method 2.2 having a lifespan of 12 frames; the best scaled scores were achieved with a lifespan of 40 frames. As it is obvious from the results in both Figure 39 and Table 5, Method 2.2 achieves very poor results with a lifespan of findings lasting 1 frame.

#### 4.1.6. Evaluation of Method 2.3

**Figure 42:**
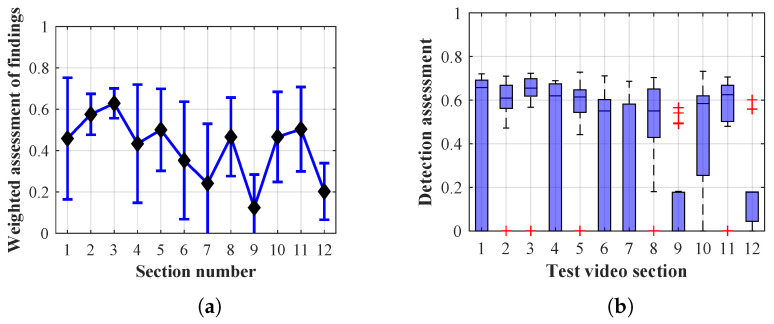
Results of Method 2.3 with a lifespan of 1 frames. (**a**) the weighted average rating with the average mean absolute error of the weighted rating and (**b**) boxplots of the distribution of the statistical sets of the results of the individual sections evaluated indicating: minimum, 1st quartile, median, 3rd quartile, maximum.

**Figure 43:**
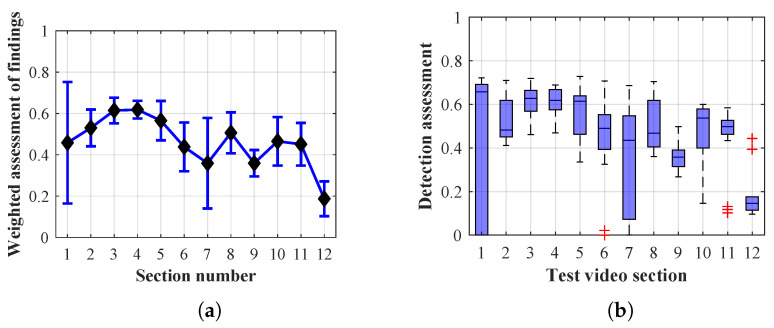
Results of Method 2.3 with a lifespan of 12 frames. (**a**) the weighted average rating with the average mean absolute error of the weighted rating and (**b**) boxplots of the distribution of the statistical sets of the results of the individual sections evaluated indicating: minimum, 1st quartile, median, 3rd quartile, maximum.

**Figure 44:**
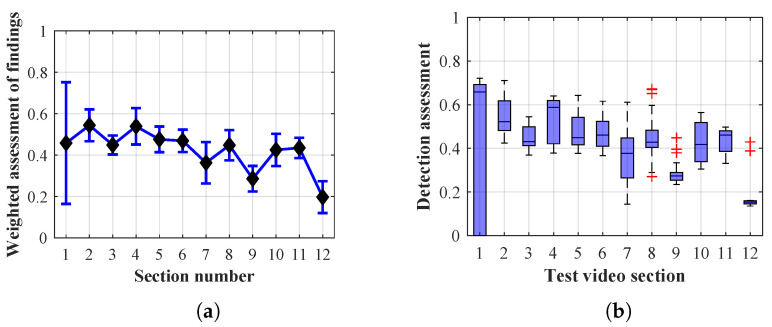
Results of Method 2.3 with a lifespan of 40 frames. (**a**) the weighted average rating with the average mean absolute error of the weighted rating and (**b**) boxplots of the distribution of the statistical sets of the results of the individual sections evaluated indicating: minimum, 1st quartile, median, 3rd quartile, maximum.

By comparing the results of Method 2.3, evaluation at a lifespan of 1, 12, and 40 frames, it is ascertained that, within the lifespan increase, an increase in the stability of the results of the weighted detection assessment in the individual sections was achieved. A graphical representation of the evaluation of the configurations used is shown, for results with a lifespan of 1 frame, in Figure 42, for results with a lifespan of 12 frames, in Figure 43, for results with a lifespan of 40 frames in Figure 44. In subfigure (b), the selected figures show boxplots of the statistical distribution of the weighted evaluation in selected sections into quartiles. The final summary of the results of all the methods in Table 5 includes the weighted average evaluation results for all the sections of the video, wherein the best weighted and scaled scores were achieved with Method 2.3 having a lifespan of 12 frames. As it is obvious from comparing the results in Figure 43 and Figure 44, Method 2.3 achieves the best score when applying a lifespan of findings of 12 frames. The error rate of the assessment increases as the lifespan extends.

#### 4.1.7. Evaluation of Method 3

**Figure 45:**
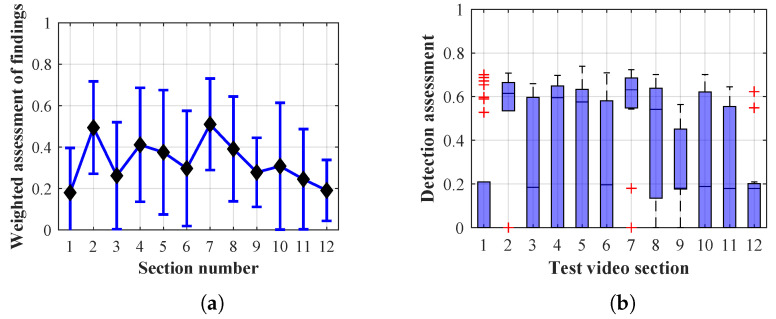
Results of Method 3 with a lifespan of 1 frames. (**a**) the weighted average rating with the average mean absolute error of the weighted rating and (**b**) boxplots of the distribution of the statistical sets of the results of the individual sections evaluated indicating: minimum, 1st quartile, median, 3rd quartile, maximum.

**Figure 46:**
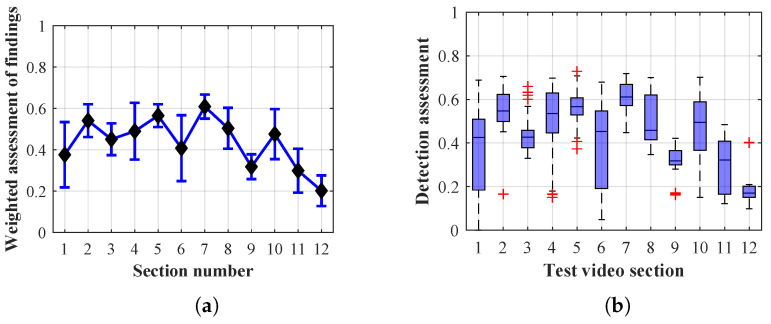
Results of Method 3 with a lifespan of 12 frames. (**a**) the weighted average rating with the average mean absolute error of the weighted rating and (**b**) boxplots of the distribution of the statistical sets of the results of the individual sections evaluated indicating: minimum, 1st quartile, median, 3rd quartile, maximum.

**Figure 47:**
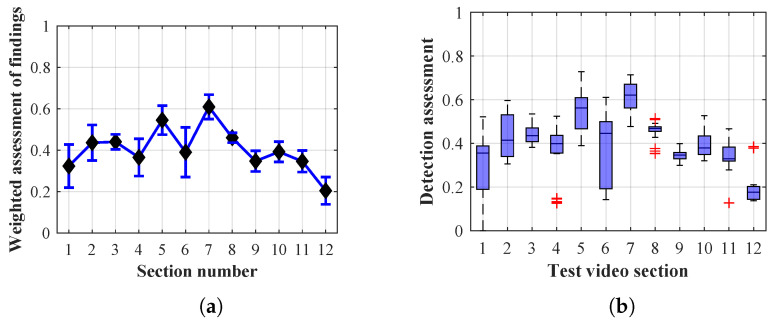
Results of Method 3 with a lifespan of 40 frames. (**a**) the weighted average rating with the average mean absolute error of the weighted rating and (**b**) boxplots of the distribution of the statistical sets of the results of the individual sections evaluated indicating: minimum, 1st quartile, median, 3rd quartile, maximum.

By comparing the results of Method 3, evaluation at a lifespan of 1, 12, and 40 frames, it is ascertained that, within the lifespan increase, an increase in the stability of the results of the weighted detection assessment in the individual sections was achieved. A graphical representation of the evaluation of the configurations used is shown, for results with a lifespan of 1 frame, in Figure 45, for results with a lifespan of 12 frames, in Figure 46, for results with a lifespan of 40 frames in Figure 47. In subfigure (b), the selected figures show boxplots of the statistical distribution of the weighted evaluation in selected sections into quartiles. The final summary of the results of all the methods in Table 5 includes the weighted average evaluation results for all the sections of the video, wherein the best weighted scores were achieved with Method 3 having a lifespan of 12 frames; the best scaled scores were achieved with a lifespan of 40 frames.

### 4.2. Mean Error Calculation

The results of the frame average assessment in the sections shown in column (a) in the figures (from Figure 27, Figure 28, Figure 29, Figure 30, Figure 31, Figure 32, Figure 33, Figure 34, Figure 35, Figure 36, Figure 37, Figure 38, Figure 39, Figure 40, Figure 41, Figure 42, Figure 43, Figure 44, Figure 45, Figure 46 and Figure 47) were plotted together with error bars having a size of $\epsilon_{i}$, both in the positive and negative direction. $\epsilon_{i}$ represents the mean error of the i-th section of the video. $\epsilon_{i}$ is calculated using the arithmetic mean ($\epsilon_{k}$) where *k* is the index of frames belonging to the i-th section. $\epsilon_{k}$ is the absolute value of the difference between $K_{i}$ and $K_{k}$ where *i* is the section index, *k* is the index of the frame being dealt with, *n* is the number of frames in the section, $K_{k}$ is the weighted rating of the selected frame and $K_{i}$ is the weighted rating of the selected section.

(5) $$K_{i}=\frac{\sum_{k=1}^{n}K_{k}}{n},$$

(6) $$\epsilon_{k}=\left|K_{i}-K_{k}\right|,$$

(7) $$\epsilon_{i}=\frac{\sum_{k=1}^{n}\epsilon_{k}}{n}.$$

### 4.3. Assessment of the Best Working Method for the Test Video Scene

Since the assessment of the results in the sections using the Saaty method is not a sufficiently transparent assessment for expressing the incorrect or correct functionality of the detection algorithm, all the results of the weighted assessment are further evaluated by the so-called scaled scores.

The limit values dividing the range of the weighted quality rating value (K) into the following intervals are:

- Lower limits of the correct finding (Q1).
- Upper limits of the incorrect finding (Q2).

(8) $$\begin{array}{c} Q_{1}=\sum_{k=1}^{n}\left(v_{i}\cdot \frac{p_{k_{i}}\mathrm{minimum}\;\mathrm{evaluated}\;\mathrm{purely}\;\mathrm{correct}\;\mathrm{finding}}{100}\right)=\;\\ 0.0351\cdot \frac{1}{100}+0.1746\cdot \frac{1}{100}+0.4626\cdot \frac{1}{100}+0.1017\cdot \frac{50}{100}+0.2260\cdot \frac{100}{100}=0.3344, \end{array}$$

(9) $$\begin{array}{c} Q_{2}=\sum_{k=1}^{n}\left(v_{i}\cdot \frac{p_{k_{i}}\mathrm{maximum}\;\mathrm{evaluated}\;\mathrm{purely}\;\mathrm{correct}\;\mathrm{finding}}{100}\right)=\;\\ 0.0351\cdot \frac{100}{100}+0.1746\cdot \frac{100}{100}+0.4626\cdot \frac{0}{100}+0.1017\cdot \frac{50}{100}+0.2260\cdot \frac{0}{100}=0.3114. \end{array}$$

Each of the assessment intervals is assigned with a value of mark γ (note: Greek letter gamma) used for the resulting calculation of the averaging detection quality of the results thus obtained. The result is a rigorous detection quality assessment that can provide better information than the previous weighted detection quality assessment relating to the success or failure of person detection in intelligent buildings using the above-mentioned system setting configurations applied.

(10) $$\left(K\geq 0\right)\wedge \left(K<Q_{2}\right)\Rightarrow \gamma =-1,$$

(11) $$\left(K\geq Q_{2}\right)\wedge \left(K<Q_{1}\right)\Rightarrow \gamma =0,$$

(12) $$\left(K\geq Q_{1}\right)\wedge \left(K\leq 1\right)\Rightarrow \gamma =1.$$

Thus, the rating (Γ) of the section of the test video containing k-frames assessed will be as follows:

(13) $$\Gamma =\frac{\sum_{i=1}^{k}\gamma_{i}}{k}.$$

The arithmetic mean of the person detection results of the individual frames at the configuration of the system of detecting persons in intelligent buildings. If the average rating (Γ) is positive or near 1, this is a good detection. If the average rating (Γ) is negative or close to 0, this is a poor detection.

Selected top-rated methods from the section comprising person detection results of the individual methods applied at different lifespans of person findings are selected from Table 5 and Table 6 as the most successful system configuration for detecting persons. In this table, the system configurations are ranked from the top-rated method to the bottom-rated method, according to the three methods for evaluating the results of the scaled scores, designated as (A), (B) and (C). Wherein score (A) is the assessment of the ranking of the results according to the average scaled scores in all sections of the video; score (B) is the assessment of the ranking of the results according to the sum of the rankings in all sections of the video; scoring (C) is the assessment of the ranking of the results according to the ranking in all sections of the video, except for the first and last section. The ranking of the methods selected thus assessed is more representative in terms of detection quality assessment because of the omission of the results from the first and last section, in which there is an unevenly increased detection error rate in all the methods due to the inconsistent presence of persons in the frames of these sections and, therefore, there is the absence of verification patterns. The best-rated method achieves mark 1; the less successful methods receive lower marks.

## 5. Conclusions

The aim of the research was to analyze and evaluate the standard and original methods of detection of the dynamic movement of persons when a person is perceived as an object moving in the scene being captured and the solution described emphasizes the appropriateness of the method providing data for the subsequent finding of a potential person in the scene being captured. Each of the original background creation solutions analyzed and the subsequent detections have their own specifics that must be taken into account and it is necessary to proceed from the research results achieved.

Appropriate weighted criteria listed in Table 3 are an important criterion for the correct evaluation of the results depending on the system requirements. The results in the final evaluation Table 6, including the best results of each of the methods from Table 5, are ranked not only according to the weighted and scaled rating in the entire range of the test video sections checked (Table 6, columns (A), (B)), but also according to the scaled rating in the range of the video sections, except for the first and last section, which resulted in significant detection errors in the rating (Table 6, column (C)). Specific methods increase errors due to detection of redundant objects in the sections, or they did not detect persons who, according to a set of verification data based on the number and possible occurrence of persons according to Table 2, do not occur in the scene being captured.

From the results shown in Table 6 (of the top-rated results), it is obvious that the top-rated configuration is the configuration of Method 1.1 having a lifespan of 12 frames, where the best-weighted score (K=0.487) was achieved compared to the worst rated method (Method 3) with the following score: K=0.328. The best-scaled rating was achieved by Method 2.2 with a lifespan of 40 frames and a result of Γ=0.789, and aforementioned Method 1.1 with a lifespan of 12 frames with a result of Γ=0.78; the worst-scaled method is Method 3 with a lifespan of 1 frame and a result of Γ=−0.015. In the attached weighted rating graphs (Figure 27, Figure 28, Figure 29, Figure 30, Figure 31, Figure 32, Figure 33, Figure 34, Figure 35, Figure 36, Figure 37, Figure 38, Figure 39, Figure 40, Figure 41, Figure 42, Figure 43, Figure 44, Figure 45, Figure 46 and Figure 47), precisely the influence and importance of using the appropriate value of the lifespan of persons, which neglects the momentary “loss” of the person found in the results of the detection of the current change in the image, are obvious. Method 1.1 with a lifespan of 12 frames is a method that uses the difference between the current image towards the static background created by the frame taken at the beginning of the test video. Errors confirming the theoretical functionality of this detection method were entered in the evaluation results (Figure 27, Figure 28 and Figure 29). These errors include longer-lasting relocation of the objects in the scene, which are then mistakenly considered as a person found; this error could be resolved by occasional restarting of the detection system, thus creating a new static background.

In this best-rated configuration, the lifespan is set to 12 frames, that is, at a frame rate of 4 fps of the test video, the lifespan of the person found is 3 seconds. Such a system setup is therefore ideal for scenes being captured where people are moving rather than acquiring static conditions in the scene.

The lowest score of the scaled rating was achieved with Method 3, which is a method that uses only memoryless optical flow. According to the results in Figure 45, Figure 46 and Figure 47, this method achieved the best detection results in section 7 shown in Table 2. This method provides information about the person detected only in pixels that are moving between the frames tested in the scene. A significant increase in the error rate was thus achieved in the test sections where no or only slight movement, insufficient for the detection of the person, was performed in the scene. This increase was compensated by the lifespan factor of the findings that were detected correctly when static conditions were acquired in the scene.

Other methods applied mentioned in Section 2 using both static and dynamic backgrounds achieved results confirming the theoretical assumptions. An important role in their application was played by the suitably selected lifespan parameter of the persons detected. As it is obvious from Figure 27, Figure 28, Figure 29, Figure 30, Figure 31, Figure 32, Figure 33, Figure 34, Figure 35, Figure 36, Figure 37, Figure 38, Figure 39, Figure 40, Figure 41, Figure 42, Figure 43, Figure 44, Figure 45, Figure 46 and Figure 47 with the plotted results of the individual sections assessment at the given system configurations, the average rating further evaluated with an error entered does not always correspond with the statistical distribution of the individual statistical files—the video sections. Outlying values are also included in the average in order to make the evaluation as strict as possible.

The research has proved experimentally and specified mathematically which of the methods is the most suitable and further applicable and usable in sophisticated computational applications implementable in intelligent buildings.

## Figures and Tables

**Table 1:** Table of currently detected findings in the scene of frame 43.

Lifespan Index	Number of Custom Pixels of the Person Found	Coordinates $x_{\min}$ Delimitation	Coordinates $x_{\max}$ Delimitation	Coordinates $y_{\min}$ Delimitation	Coordinates $y_{\max}$ Delimitation
4	4743	281	413	76	138
3	38,632	3	283	160	404

**Table 2:** Description of test video analysis sections.

Video Section	Section Interval (Video Frames)	Actions in the Section
1	1 to 6	empty room with no movement
	7 to 30	door opening, entrance of person no.1, door closing
2	31 to 40	person no.1—walking across the room
	41 to 48	person no.1—grabbing an object (a folder on the bed)
3	49 to 70	person no.1—sitting down, end of movement
4	71 to 73	person no.1—at rest
	74 to 84	person no.1—at rest, door opening, entrance of person no.2
	87 to 96	person no.1—at rest, door opening, entrance of person no.2
5	97 to 134	person no.1—at rest, person no.2—shifting a chair and sitting down
	135 to 142	person no.1—at rest, person no.2—at rest
6	143 to 161	person no.1—walking across the room, person no.2—at rest
	162 to 172	person no.1—walking across the room, person no.2—at rest,
		intersecting of persons in the image
	173 to 188	person no.1—at rest, person no.2—at rest,
		intersecting of persons in the image
	189 to 190	person no.1—walking across the room, person no.2—at rest,
		intersecting of persons in the image
7	191 to 215	person no.1—walking across the room, person no.2—at rest
	216 to 229	person no.1—pushing a toy car, person no.2—at rest
	230 to 239	person no.1—at rest, person no.2—at rest
8	240 to 268	person no.1—at rest, person no.2—walking across the room
	269 to 272	person no.1—at rest, person no.2—at rest
9	273 to 281	person no.1—at rest, person no.2—at rest,
		darkening of the room using blinds
	282 to 296	person no.1—at rest, person no.2—at rest,
		brightening of the room using blinds
10	297 to 319	person no.1—walking across the room (exit), person no.2—at rest
11	320 to 339	person no.2—walking across the room (exit)
12	340 to 346	door closing
	347 to 350	empty room with no movement

**Table 3:** Saaty matrix with geometric means calculated, and standardized weights of the individual criteria.

	$K_{1}$	$K_{2}$	$K_{3}$	$K_{4}$	$K_{5}$	$G_{i}$	$v_{i}$
$K_{1}$	1	0.2	0.111	0.333	0.111	0.241	**0.0351**
$K_{2}$	5	1	0.166	3	1	1.200	**0.1746**
$K_{3}$	9	6	1	3	2	3.178	**0.4626**
$K_{4}$	3	0.333	0.333	1	0.5	0.699	**0.1017**
$K_{5}$	9	1	0.5	2	1	1.552	**0.2260**
						6.87	1

**Table 4:** The results of the weighted person detection assessment using Method 1.1.

Section Number	Weighted Average Rating			The Mean Error of Measurement Rating		
	Lifespan of 1 Frame	Lifespan of 12 Frames	Lifespan of 40 Frames	Lifespan of 1 Frame	Lifespan of 12 Frames	Lifespan of 40 Frames
1	0.477	0.477	0.477	0.294	0.294	0.294
2	0.623	0.609	0.610	0.056	0.049	0.048
3	0.629	0.551	0.506	0.017	0.075	0.024
4	0.626	0.620	0.541	0.030	0.038	0.058
5	0.555	0.552	0.553	0.030	0.027	0.029
6	0.459	0.487	0.488	0.077	0.045	0.044
7	0.504	0.493	0.497	0.040	0.030	0.029
8	0.505	0.478	0.479	0.028	0.030	0.030
9	0.337	0.411	0.424	0.156	0.059	0.062
10	0.469	0.475	0.391	0.071	0.056	0.040
11	0.326	0.456	0.432	0.202	0.096	0.120
12	0.262	0.232	0.245	0.091	0.102	0.097
Average of rating of all sections	0.481	0.487	0.470	0.091	0.075	0.073
Final ranking, from top-rated	2	1	3			

**Table 5:** Table of sorting the tested system configurations according to the average scaled rating in the test video sections, from top-rated.

System Configuration Used (Change Detection Method and Lifespan of Person Finding)	Result of the Weighted Rating—K	Result of the Scaled Rating—Γ	Ranking of the Results According to the Average Scaled Rating in all Sections of the Test Video
Method 1.1 with a lifespan of 1 frame	0.481	0.650	9
Method 1.1 with a lifespan of 12 frames	**0.487**	**0.780**	2
Method 1.1 with a lifespan of 40 frames	0.470	0.775	4
Method 1.2 with a lifespan of 1 frame	0.473	0.622	13
Method 1.2 with a lifespan of 12 frames	**0.476**	**0.762**	6
Method 1.2 with a lifespan of 40 frames	0.460	0.747	7
Method 1.3 with a lifespan of 1 frame	0.479	0.643	10
Method 1.3 with a lifespan of 12 frames	**0.485**	0.772	5
Method 1.3 with a lifespan of 40 frames	0.469	**0.776**	3
Method 2.1 with a lifespan of 1 frame	0.400	0.215	20
Method 2.1 with a lifespan of 12 frames	**0.467**	0.574	15
Method 2.1 with a lifespan of 40 frames	0.438	**0.639**	11
Method 2.2 with a lifespan of 1 frame	0.432	0.349	18
Method 2.2 with a lifespan of 12 frames	**0.468**	0.715	8
Method 2.2 with a lifespan of 40 frames	0.453	**0.789**	1
Method 2.3 with a lifespan of 1 frame	0.412	0.278	19
Method 2.3 with a lifespan of 12 frames	**0.463**	**0.623**	12
Method 2.3 with a lifespan of 40 frames	0.424	0.552	16
Method 3 with a lifespan of 1 frame	0.328	-0.015	21
Method 3 with a lifespan of 12 frames	**0.437**	0.539	17
Method 3 with a lifespan of 40 frames	0.405	**0.608**	14

**Table 6:** Table ranking the best results (A) Ranking of the results according to the average scaled rating in all sections of the video; (B) Ranking of the results according to the rankings in all sections of the video; (B) Ranking of the results according to the rankings in all sections of the video, except for the first and last section.

System Configuration Used	(A)	(B)	(C)
Method 1.1 with a lifespan of 12 frames	2	1	1
Method 1.3 with a lifespan of 12 frames	3	2	2
Method 2.2 with a lifespan of 40 frames	1	4	3
Method 1.2 with a lifespan of 12 frames	4	3	4
Method 2.3 with a lifespan of 12 frames	6	5	5
Method 2.1 with a lifespan of 40 frames	5	6	6
Method 3 with a lifespan of 40 frames	7	7	7
